# In vitro sexual dimorphism establishment in schistosomes

**DOI:** 10.7554/eLife.111066

**Published:** 2026-09-10

**Authors:** Rémi Pichon, Magda E Lotkowska, Jude LD Bulathsinghalage, Madeleine McMath, Mary Evans, Benjamin J Hulme, Kirsty Ambridge, Geetha Sankaranarayanan, Simon Kershenbaum, Sarah D Davey, Josephine E Forde-Thomas, Karl F Hoffmann, Matthew Berriman, Gabriel Rinaldi

**Affiliations:** 1 Department of Biology, University of Oxford Oxford United Kingdom; 2 Department of Life Sciences, Aberystwyth University Aberystwyth United Kingdom; 3 Wellcome Sanger Institute Hinxton United Kingdom; 4 School of Infection and Immunity, College of Medical, Veterinary & Life Sciences, University of Glasgow Glasgow United Kingdom; University of Geneva Switzerland; University of Geneva Switzerland

**Keywords:** schistosomiasis, *Schistosoma* spp, long-term culture, human serum, in vitro schistosome development, sexual dimorphism, Other

## Abstract

Schistosomes are parasitic flatworms that cause Schistosomiasis, a major neglected tropical disease that affects more than 250 million people worldwide. With two distinct sexes, a heterogametic female (ZW) and a homogametic male (ZZ), schistosomes are an exception among flatworms, which are largely hermaphroditic. Sexual dimorphism in schistosomes only becomes apparent by adulthood within the mammalian host. However, the cellular and molecular mechanisms underlying the sexual differentiation of are poorly understood, partly due to intrinsic challenges in assessing parasite development in vivo. Therefore, robust and reproducible approaches for maintaining and developing parasites in vitro are needed to overcome these difficulties. To date, few studies have focused on protocols that allow cultured parasites to reach sexual dimorphic stages, and none have been reproduced, limiting the ability to understand the sexual biology of this major human parasite. Here, we refine a protocol for long-term culture of newly transformed cercariae that developed in vitro into sexually dimorphic forms. We assessed the effect of adding two different sera, foetal bovine serium (FBS) and human serum (HS), to culture medium supplemented with red blood cells. In contrast to FBS-culture parasites, those grown in HS digested red blood cells, a crucial step for long term parasite development. Furthermore, sexual dimorphism was clearly established in the HS-cultured parasites, albeit delayed, in contrast to most FBS-cultured parasites that did not progress beyond an early liver stage. Moreover, in EdU-pulse experiments, cells within HS-cultured parasites continuously proliferated, but markedly fewer proliferated in FBS-culture. By enabling reproducible parasite develoment in vitro, this protocol creates new opportunities for dissecting mechanisms that underly sexual dimorphim, as well as for screening in vitro for new interventions across the life cycle of these major human parasites.

## Introduction

Schistosomiasis, a major neglected tropical disease (NTD) affecting >250 million people worldwide, is caused by the infection with blood flukes (class Trematoda) in the genus *Schistosoma* (LoVerde, 2024). The pathology associated with schistosomiasis is largely driven by egg trapping in different tissues depending on the species. Infection with *Schistosoma mansoni* leads to eggs lodged mainly in the liver, inducing inflation, fibrosis, and granuloma formation (Buonfrate et al., 2025). This suggests that interfering with the sexual development of schistosome intra-mammalian stages could potentially restrict human pathology. Currently, the complete reliance on a single drug (Praziquantel) to treat the infection and its use in drug mass administration programmes in endemic areas threatens the development of drug resistance (Berger et al., 2024; Crellen et al., 2016). Therefore, novel control strategies are urgently needed, and identifying original targets for drug/vaccine development became a priority. A better understanding of the mechanisms underlying schistosome development, including sexual dimorphism establishment, will pave the way to achieve this goal.

While most parasitic flatworms are hermaphrodites, schistosomes are dioecious with genetically determined female (2n=16, ZW) and male (2n=16, ZZ) individuals. Whilst male and female parasites are morphologically indistinguishable across most developmental stages, sexual dimorphism becomes apparent by adulthood within the mammalian host (Loker and Brant, 2006). Male and female worms undergo separate but concurrent sexual differentiation of their gonads and somatic tissues that eventually allows intersexual pairing, a critical step for female maturation, egg production, and life cycle propagation (Chen et al., 2022; Shakir et al., 2023). Transcriptomic studies, at both bulk (Elkrewi et al., 2021; Fitzpatrick and Hoffmann, 2006; Lu et al., 2016; Picard et al., 2016; Wangwiwatsin et al., 2020) and single cell (Diaz Soria et al., 2020; Li et al., 2021; Wendt et al., 2020) levels for intra-mammalian stages in vivo and ex vivo, have revealed key pathways and molecular mediators underlying the critical process of female sexual maturation induced by pairing. However, the cellular and molecular mechanisms driving sexual dimorphism prior to pairing remain poorly characterised, partly due to intrinsic challenges in assessing the development of the parasite in vivo (Wangwiwatsin et al., 2020).

Approaches to better understand the biology of parasites at large have been developed and optimised using in vitro or ex vivo refined culture systems (Pance and Rinaldi, 2024; Sutrave and Richter, 2023). Combinations of culture media components, sera from different sources and additives have been tested (Ahmed, 2014; Mann et al., 2010). In addition, *organ-on-a-chip* systems and organoid-based 2D and 3D culture platforms Mellin and Boddey, 2020; Zorrinho-Almeida et al., 2025 have first been implemented for protozoa parasites such as *Plasmodium*, *Toxoplasma*, and *Cryptosporidium* species. These methods have facilitated the molecular dissection of processes underlying single-cell parasite development and interaction with the host (Faral-Tello et al., 2023; Korwin-Mihavics et al., 2023; Zhou et al., 2024). Recently, these technologies have started to be transferred to metazoan parasites to study host–parasite interaction and immune response in the context of helminth infections (Britton et al., 2023; Duque-Correa et al., 2022; Faber et al., 2022; Vitkauskaite et al., 2025; White et al., 2022). In vitro cultivation of both larval tapeworms (cestodes) and stem cells isolated from cestodes, coupled with ‘omic’ and imaging analyses, have unveiled the role of stem cells in parasite development (Herz et al., 2024; Koziol and Brehm, 2015). However, despite these significant developments in the field, reproducing the natural development of trematodes under controlled in vitro conditions remains extremely challenging (Sutrave and Richter, 2023).

Significant progress was achieved in culturing schistosomes when, more than 40 years ago, Paul F. Basch developed and optimised comprehensive culture protocols for intra-mammalian developmental stages of schistosomes (Basch, 1981b; Basch, 1981a; Basch and Humbert, 1981c). Basch reported for the first time the development of male and female parasites from cercariae, with worm pairing and production of infertile eggs entirely in vitro (Basch, 1981b). Remarkably, to the best of our knowledge, these results have not been replicated and no further attempt to obtain egg-laying worm pairs developed in vitro from cercariae has been reported since Basch’s seminal studies (Sutrave and Richter, 2023). More recently, in vitro culture protocols for schistosomes have been refined either to maintain ex vivo parasites collected from infected mice to study parasite reproduction biology (Wang et al., 2019; You et al., 2024), or to culture juvenile parasites from cercariae and develop drug screening approaches (Frahm et al., 2021; Buchter et al., 2021; Maharjan et al., 2021). However, no long-term culture (LTC) conditions to specifically assess schistosome sexual differentiation have been reported since 1981 (Basch, 1981a). Despite one research group referring to the in-vitro development of egg-laying adult worms from cercariae (Abla et al., 2017), there has been no detailed description and no widespread adoption. In fact, published culturing studies have continued to utilise foetal bovine serum (FBS) (Wang et al., 2019; Milligan and Jolly, 2011).

Novel functional approaches applied to culture systems that allow reliable and reproducible in vitro establishment of sexual dimorphism will shine new light into molecular mechanisms underlying schistosome intra-mammalian development. We aimed at optimising a platform to study intra-mammalian schistosomes that supports in vitro sexual dimorphism establishment and consequently leading to an overall positive impact in the 3Rs (reduction, replacement, and refinement) on animal research (https://nc3rs.org.uk/) (Louis-Maerten et al., 2024). Here, we refined a protocol for LTC of newly transformed cercariae by assessing the effects of two different sera, FBS and human serum (HS), added to the medium supplemented with human red blood cells (hRBCs). Striking differences were evident between parasites maintained in either FBS or HS. First, phenotypic differences between FBS- and HS-cultured parasites became evident as early as 48 hr in culture, with HS-cultured parasites exhibiting higher rates of cell proliferation resulting in larger worms in the HS condition. Second, hRBC were digested and hemozoin became apparent in the intestines of HS-cultured parasites within a day in contrast to FBS-cultured parasites that mostly lacked visible hemozoin. Finally, while most of the FBS-cultured parasites did not progress beyond lung and early liver stage, HS-cultured parasites reached sexually dimorphic stages by week 6, albeit at a slightly delayed rate compared to in vivo development. In the mouse model, parasites become dimorphic by day 21 post-infection (~3 weeks; Wangwiwatsin et al., 2020). Taken together, this protocol allows early sexual development of schistosomes to be studied in vitro, provides a method for high-throughput drug screening targeting parasite development, and reduces the reliance on animals in research.

## Results

### Sexually dimorphic schistosomes developed entirely in vitro from cercariae

**Figure 1.**
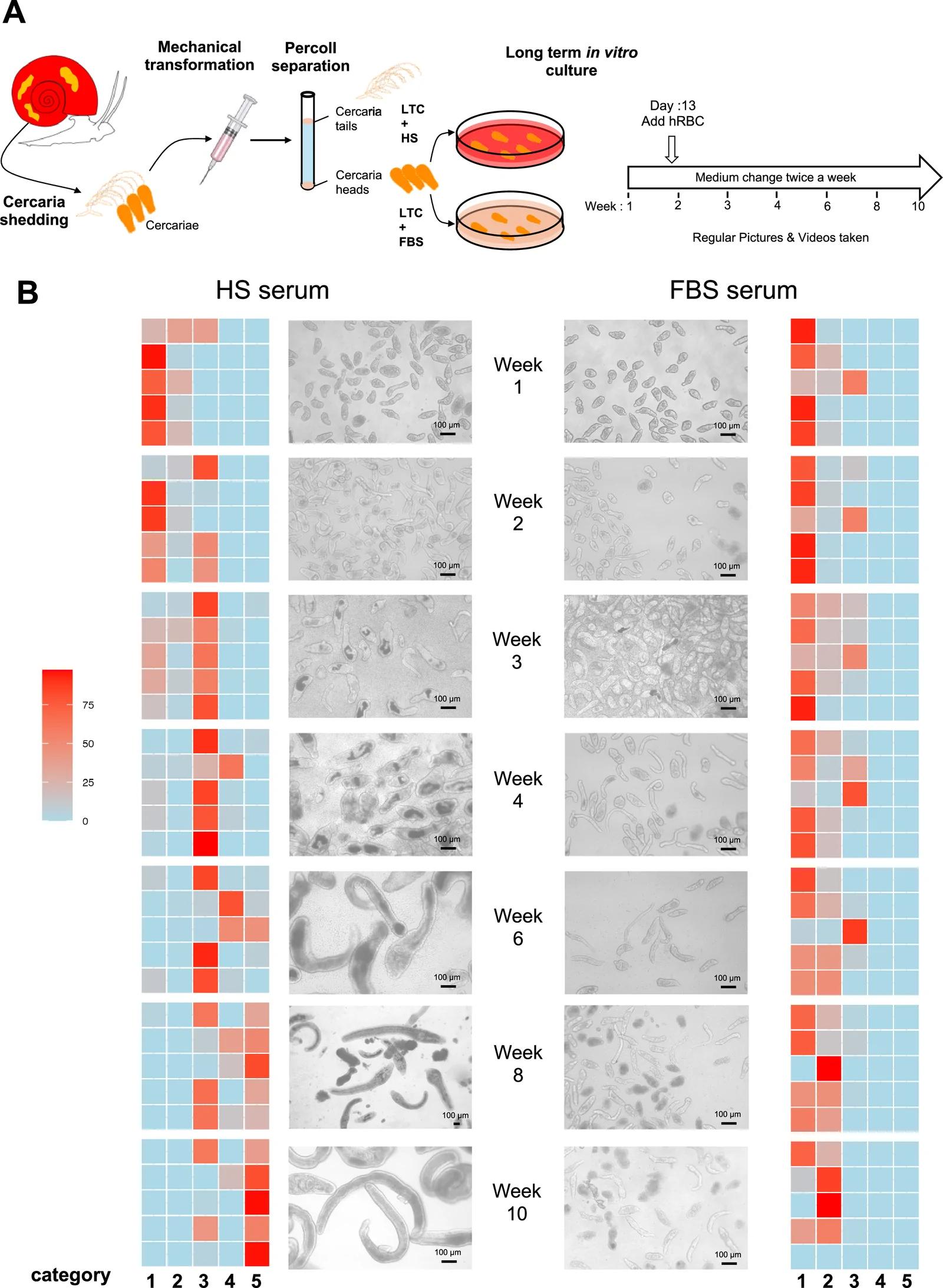
Dimorphic female and male schistosomes entirely developed in vitro from cercariae. (**A**) Schematic representation of the collection and mechanical transformation of cercariae into schistosomula for long-term in vitro culture. (**B**) Morphological scoring of cultured *S. mansoni* schistosomula at the indicated time points after in vitro transformation (weeks 1–10) for worms in long-term culture (LTC) medium supplemented either with human serum (HS - left) or foetal bovine serum (FBS - right). Heatmap columns represent five distinct morphological categories, and rows indicate five independent culture experiments, that is parasites obtained from different batches of infected snails. Heatmap colours represent the percentage of worms for each replicate in each morphological category. Middle panel: representative images of in vitro schistosomula cultured in either HS or FBS as indicated. Scale bars: 100 µm. Category 1, early schistosomula; category 2, lung schistosomula; category 3, early liver schistosomula; category 4, late liver schistosomula; category 5, dimorphic schistosomula. A detailed description of the developmental categories and representative images are provided in Figure 1—figure supplement 1.

Searching for culture media able to cultivate parasites from cercariae to maturity, we decided to test HS compared to the commonly used FBS. This rationale was based both on the fact that humans are the definitive host of *S. mansoni*, and that two previous reports in 1981 showed that HS was capable of producing mature schistosomes in vitro (Basch, 1981b; Basch, 1981a), but they were never fully replicated. More recently, culture media were complemented with HS in studies focused on early stages of parasite development for drug testing (Frahm et al., 2021; Buchter et al., 2021; Maharjan et al., 2021); however, these worms did not progress beyond the liver stage (Frahm et al., 2019). Therefore, to ascertain the in vitro sexual dimorphism establishment of schistosomes entirely developed from cercariae, we compared the effect of two different sources of blood serum: FBS and HS. The development of schistosomula derived from mechanically transformed cercariae was assessed in at least 15 independent experiments, five of which were maintained over a period of at least 10 weeks to assess parasite survival and ability to mate and produce fertile eggs (Figure 1A; Supplementary file 1A).

**Figure 1—figure supplement 1.**
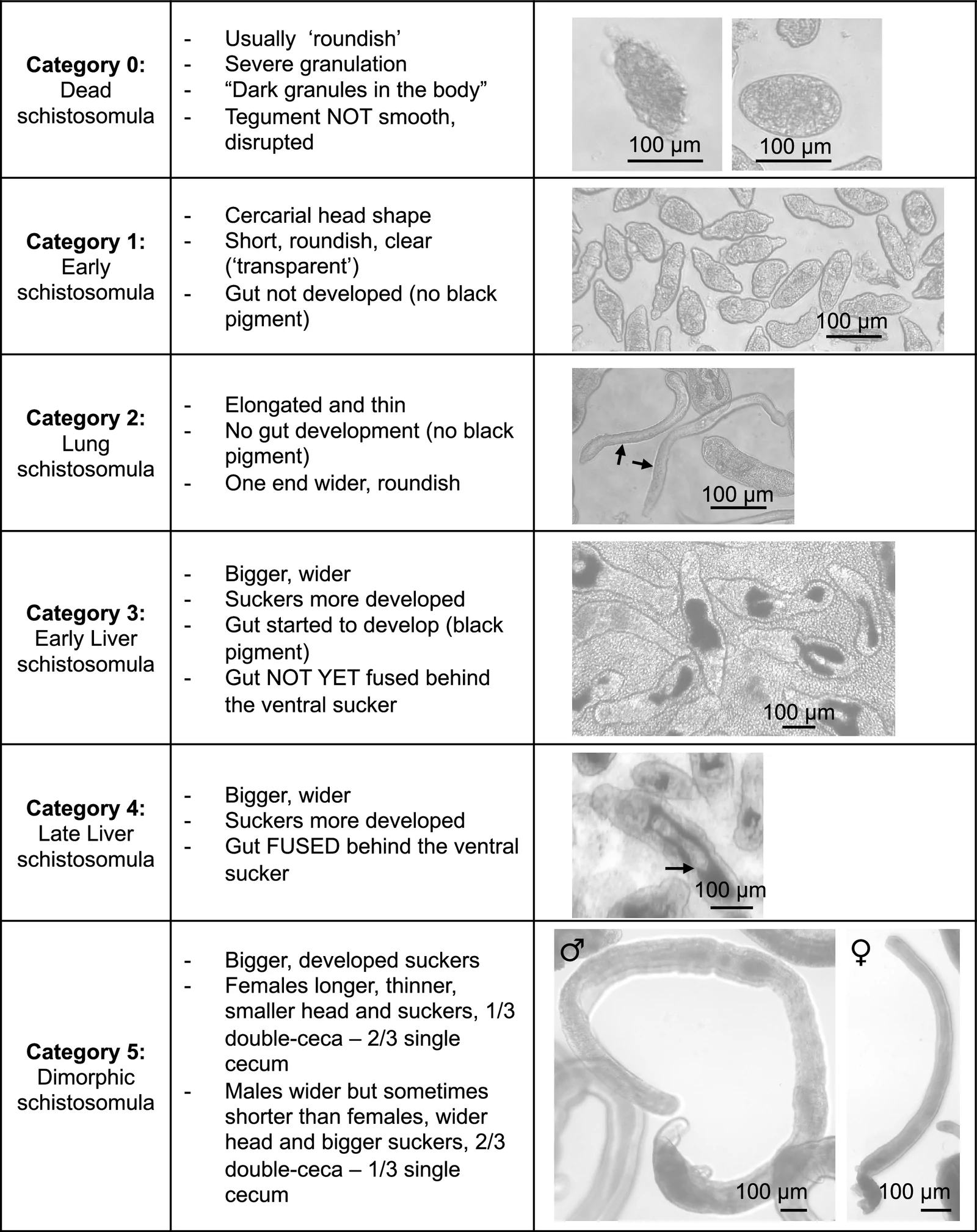
Developmental stage scoring. Summary of morphological attributes for the five different categories of development of worms cultivated in vitro (HS), as well as representative bright field images of each of these morphological categories. Arrows in categories 2 and 4 indicate elongated lung schistosomula, and the fusion of the two caeca posterior to the ventral sucker in the late liver schistosomula, respectively. Scale bar: 100 µm.

**Figure 1—figure supplement 2.**
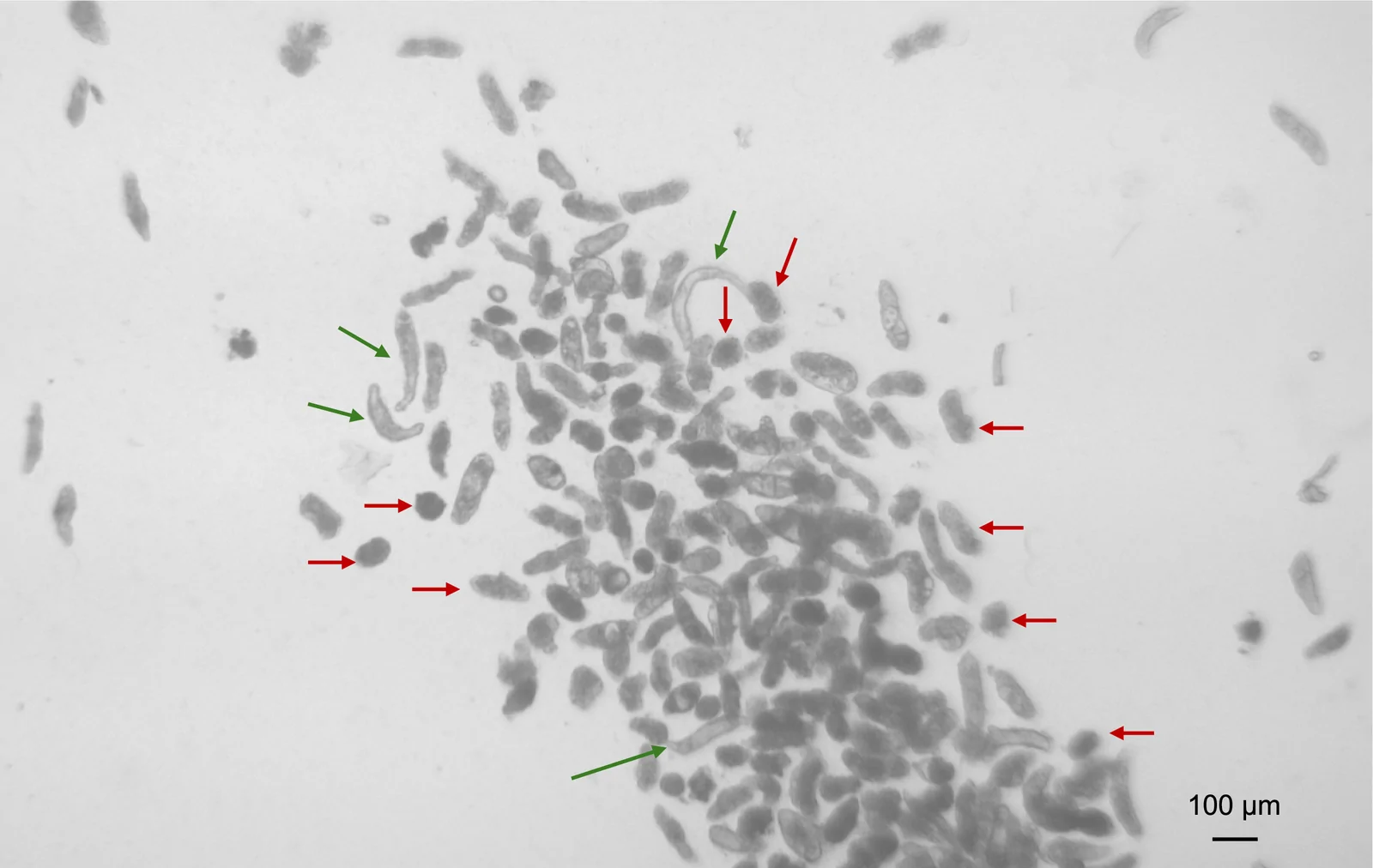
Representative FBS-cultured parasites by week 10 in culture; most of the worms are dead (red arrows) and the few still alive are lung schistosomula (green arrows). Scale bar: 100 µm.

**Figure 1—figure supplement 3.**
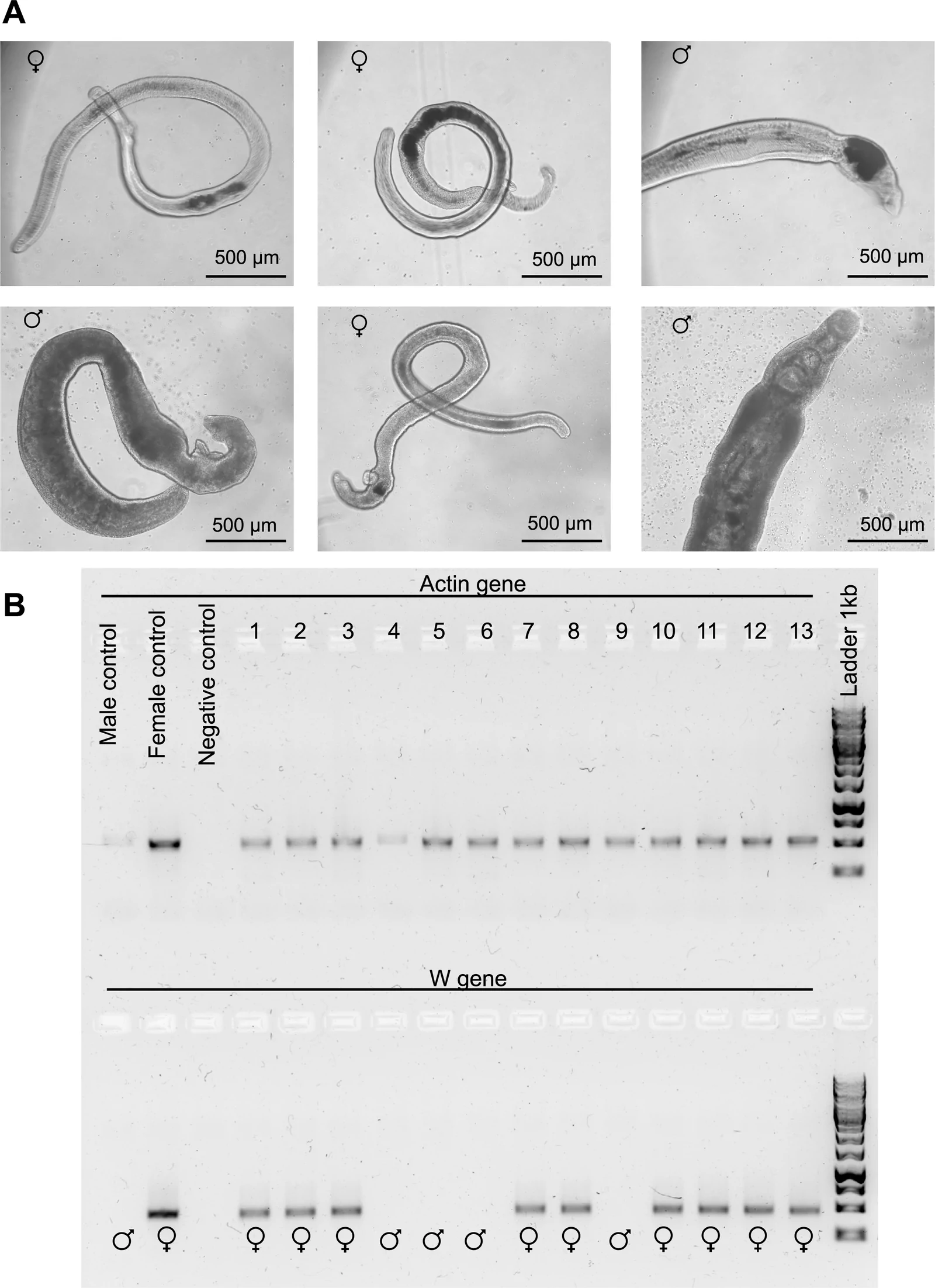
Dimoprhic worms developed in vitro. (**A**) Representative pictures of in vitro developed parasites by day ~80 in culture, with male or female worms as indicated. Scale bar: 500 µm (**B**) Representative results of the PCR-based sex genotyping using primers to amplify a fragment of the control actin gene in both male and female (upper band), and primers to amplify a W-specific region only in female worms (lower band). A non-template control, and male and female positive controls were included as indicated. Assigned sexes for each of the groups of clonal cercariae emitted by individual snails infected with single miracidia.

No morphological differences were observed between parasites cultured either in FBS or HS within the first week in culture; in both conditions, most parasites were classified as early schistosomula (category 1: 76%±30 [average ± SD] in FBS and 73%±29 [average ± SD] in HS) with few lung (category 2) and early liver schistosomula (category 3; Figure 1B, week 1; Figure 1—figure supplement 1). The mean mortality (category 0) at week 1 was slightly higher, but not statistically significant (p=0.42), in worms cultured in HS (9.75%±2.76 [average ± SD]) compared to the mortality registered in FBS-cultured parasites (5.52%±5.18 [average ± SD], Figure 1—figure supplement 2; Supplementary file 1B), consistent with previous findings (Maharjan et al., 2021).

Differences in parasite development between the two conditions became apparent by week 2 (Figure 1B). At this time point, 14.8%±24.9 (average ± SD, excluding dead worms) or 36%±33.6 (average ± SD, excluding dead worms) of the parasites cultured in FBS or HS, respectively, have reached category 3, that is early liver schistosomulum. Parasites in FBS rarely progressed beyond this stage during the 10-week experiment, with very few parasites (<0.1% ± 0.2, average ± SD) reaching category 4, that is late liver schistosomulum. In contrast, worms cultured in HS developed over time across all categories, achieving marked sexual dimorphism by week 6 (13.4%±18.6, average ± SD; Figure 1B; Figure 1—figure supplement 3), as confirmed by PCR (Figure 1—figure supplement 3; Supplementary file 1C). No differences in the timing for sexual dimorphism establishment were observed between male and female parasites. The mortality rate of FBS-cultured parasites reached an average of 76.24%±23.46 (average ± SD) by week 10, after which the experiments under this condition were stopped as most parasites were dead (Figure 1—figure supplement 2). From that time point onwards only parasites in HS were kept in culture. As previously described for the in vivo development of schistosomes (Wangwiwatsin et al., 2020), in vitro cultured parasites showed developmental asynchrony in agreement with Basch’s observations (Basch, 1981a); however, by week 10 most of the worms in HS (73.7%±25.4, average ± SD) acquired an evident sexual dimorphism (Figure 1B).

**Figure 2.**
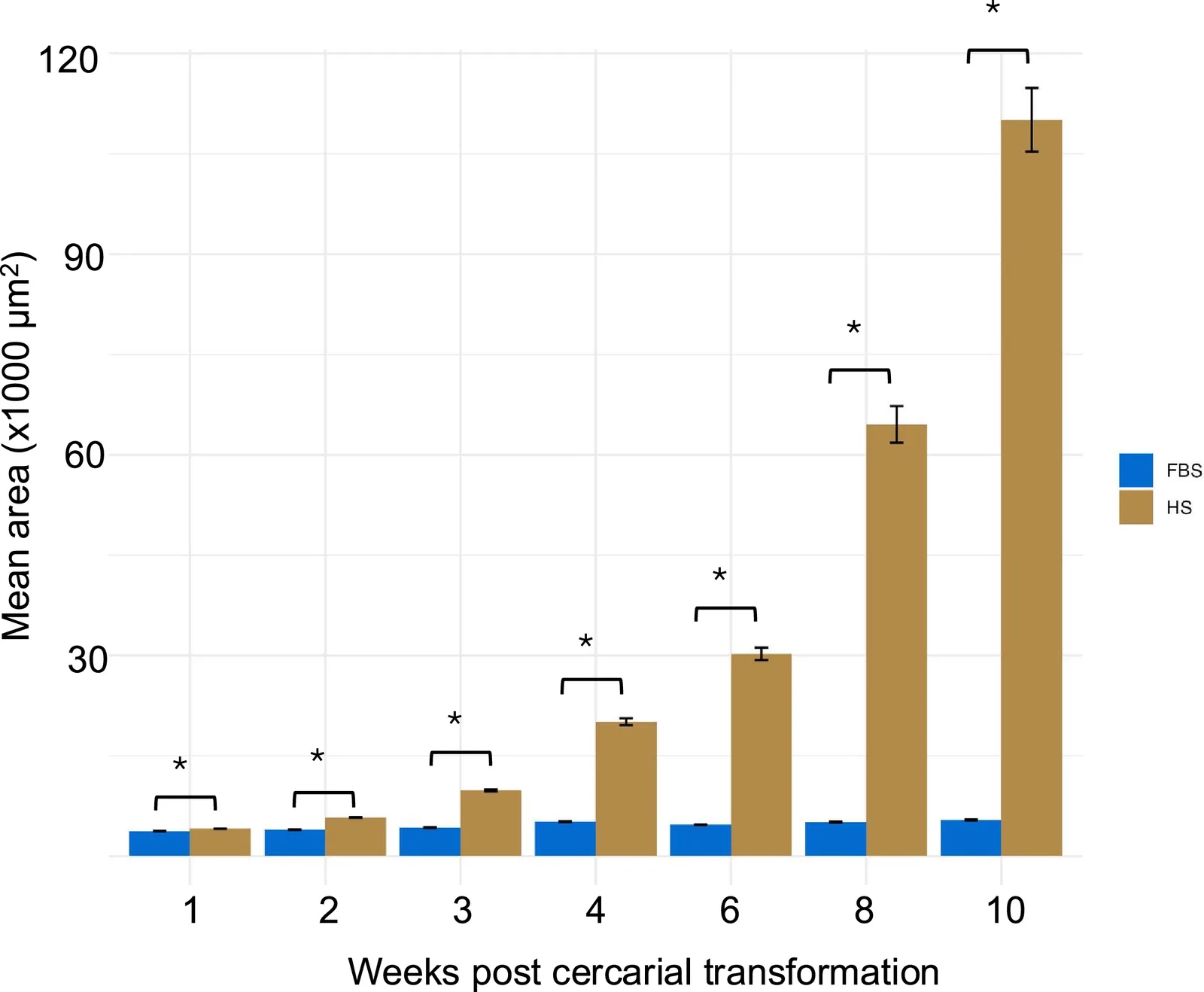
Parasites cultured in human serum (HS) grew in size unlike those cultured in foetal bovine serum (FBS). Bar plot representing area measurements of schistosomula developed in vitro and cultured in media complemented either in FBS or HS at indicated weeks after cercarial transformation. Bars indicate mean area (µm²) ± SEM. HS (light brown); FBS (blue). *: p-value <0.01 in indicated pairwise comparisons (Supplementary file 1F).

The differences in parasite development between the two tested conditions were also confirmed by measuring worm areas at different time points in culture (Figure 2; Supplementary file 1D). Parasites cultured in the presence of HS grew exponentially over time to reach on average 110,069 μm^2^±72,959 (average ± SD), ~20-fold larger than parasites cultured in FBS for 10 weeks, which only grew slightly, plateauing at an average 5417 μm^2^±1,322 (average ± SD), similar to the size reached by parasites after only 2 weeks in culture with HS (p-value <0.01, Supplementary file 1B; Figures 1 and 2).

### Parasites developed in human serum readily digest red blood cells

**Figure 3.**
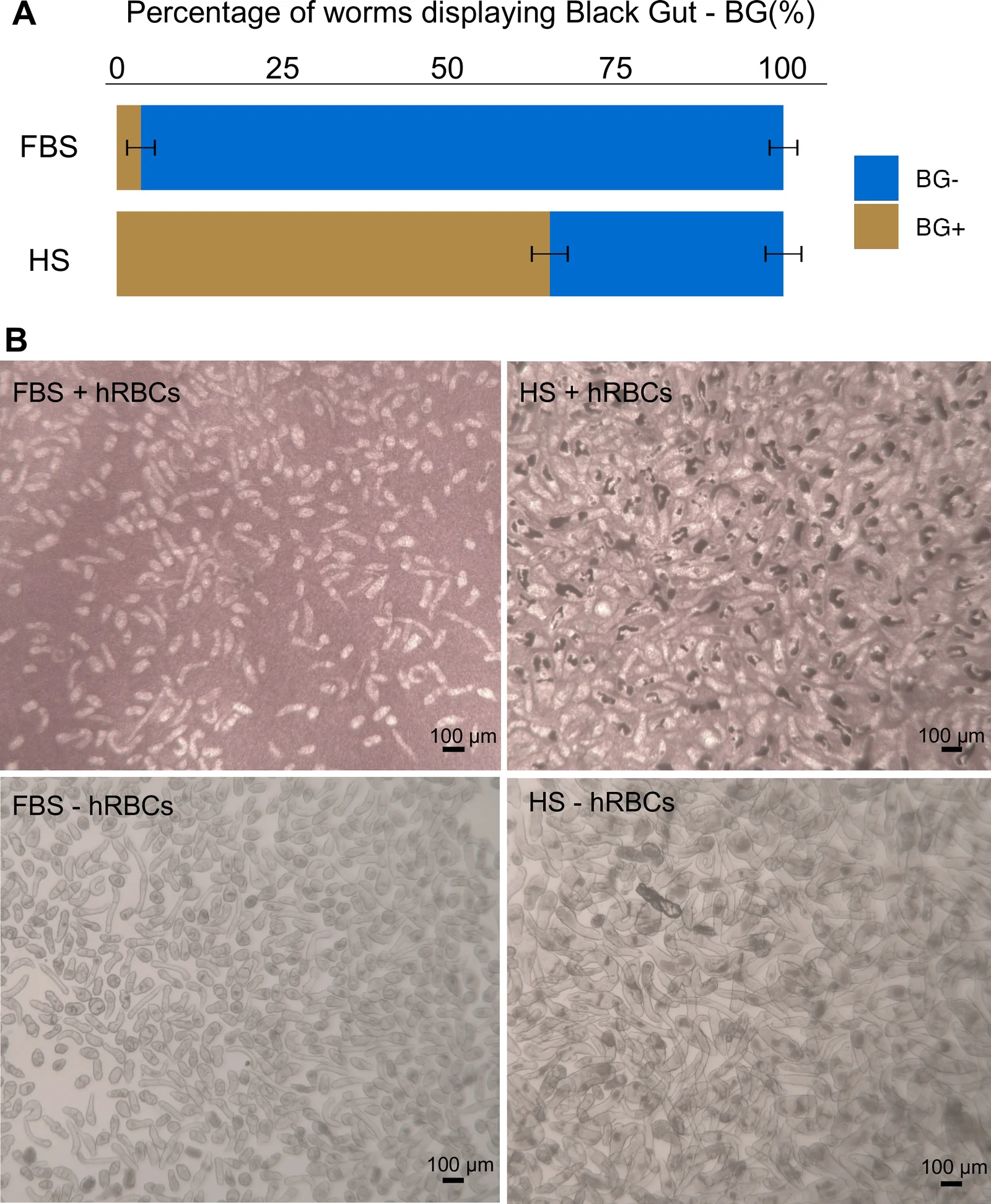
Parasites developed in human serum (HS) readily digest RBCs. (**A**) Bar Plot representing the percentage of HS- or foetal bovine serum (FBS)-cultured schistosomula with (BG+, light brown bar) or without (BG-, blue bar) black guts (BG) due to the presence of intestinal hemozoin. Washed human red blood cells (hRBCs) were added into the media at day 13 post-transformation and images captured 1 or 2 days later. Error bars = SEM. (Statistical analyses in Supplementary file 1F). (**B**) Representative images of in vitro developed schistosomula cultured in FBS or HS 1 day after adding hRBC (+RBC) and controls without RBC (- RBC). Scale bars: 100 µm.

It is widely accepted that within the mammalian host, schistosomes begin to feed on blood cells through their digestive tract at 10 days post-infection; at this time, the majority of the parasites have left the lungs and started to colonise the portal system veins in the liver (Nation et al., 2020). Based on both previous reports (Correnti et al., 2007) and pilot experiments in which adding hRBCs to the culture before day ~10 did not show obvious haemoglobin digestion, we decided to supplement the culture media with hRBCs at day 13. The addition of hRBCs allowed the parasites to feed and thus continue their development (Mann et al., 2010). At this point, they began to swallow and degrade erythrocytes, producing hemozoin, a black pigment derived from host haemoglobin degradation and visible in the worms' intestines. Even though few parasites in FBS reached the early liver stage (category 3) within the first week in culture, a minority of them were able to digest hRBCs (3.6%±4.7, average ± SD), indicated by displaying black guts (BG; Figure 3). In contrast, a significant proportion of HS-cultured parasites (36.2%±33.6, average ± SD) had already reached the early liver stage by the second week in three out of five experiments (Figure 1B, week 2). Moreover, parasites cultured in HS displayed a functional digestive system capable of assimilating hRBCs; more than half of the HS-cultured parasites (65%±6, average ± SD, p-value <0.05) showed BG in comparison to FBS-cultured parasites (3.7 ± 4.7 %; Figure 3; Supplementary file 1E).

### Development of parasites in human serum may be driven by stem cell proliferation

**Figure 4.**
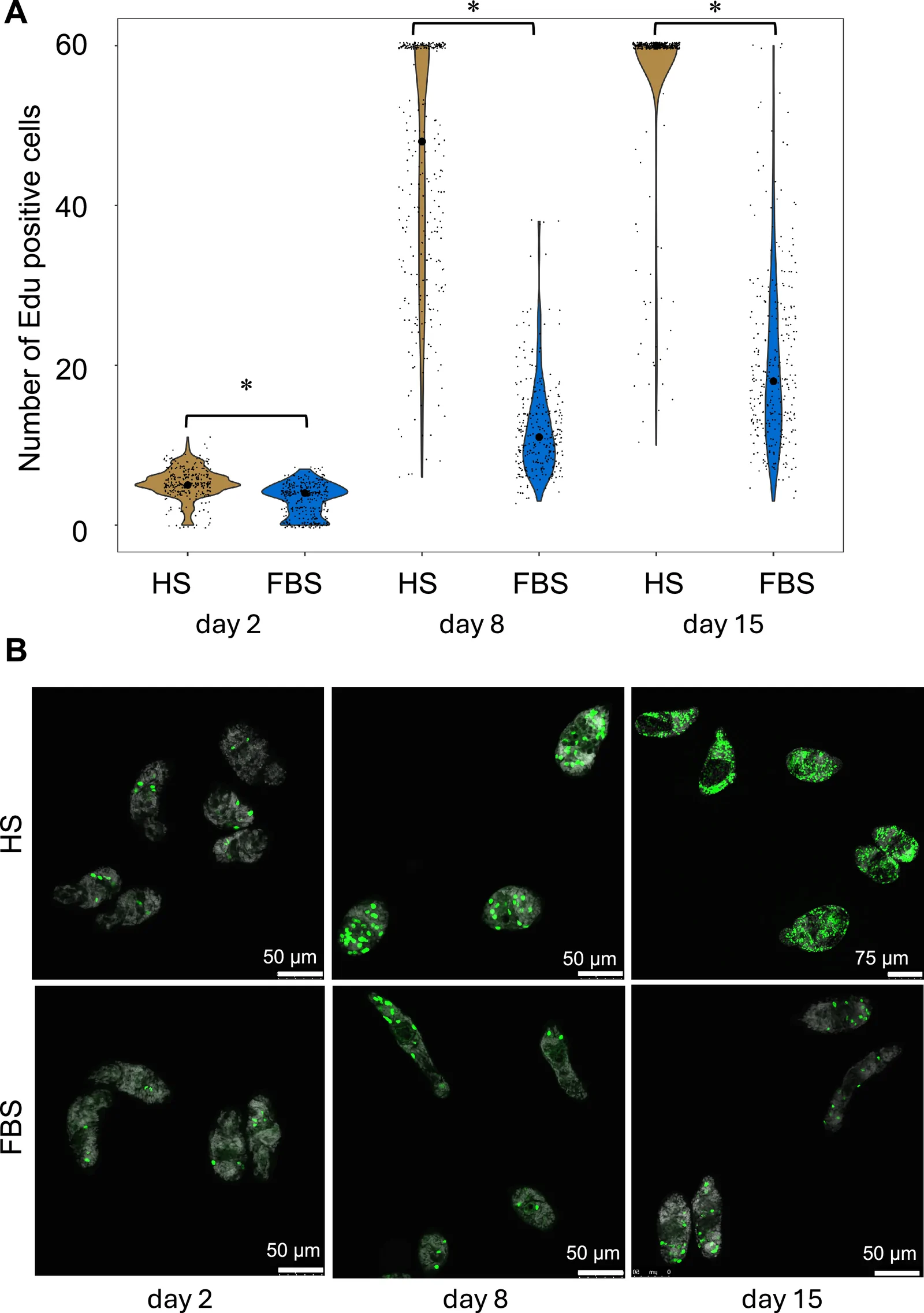
Development of parasites in human serum (HS) may be driven by stem cell proliferation. (**A**) Violin plots showing the number of EdU + cells per worm at indicated time points (2, 8, and 15 days post-cercarial transformation) in parasites cultured either in foetal bovine serum (FBS, blue) or HS (light brown). Human red blood cells (hRBCs) were added in the culture at day 13 post-cercarial transformation. The small black dots indicate individual worms, and the big black point indicates the median of EdU + cells per worm. All worms showing ⪰ 60 EdU+ cells were counted and clustered together in the group named ‘60 EdU + cells’. Hence, the data were treated as ordinal, and statistical analysis performed by Kruskal–Wallis test with Dunn multiple comparison post-hoc test, with p≤0.05 (*) considered significant (Supplementary file 1E and F). (**B**) Representative images of parasites displaying Edu + cells at each indicated time point and culture condition. Edu + cells and nuclei were labelled with Alexa fluor 488 (green) and DAPI (white/grey), respectively. Scale bars: 50 µm or 75 µm as indicated.

The growth and development of an organism is driven by a finely regulated combination of cell hyperplasia, hypertrophy, and apoptosis across different tissues (Canepa et al., 2024). In schistosomes, a complex stem cell system consisting of both somatic and germline stem cells has been described by leveraging recent single-cell transcriptomic data across different developmental stages, including schistosomula and adult worms (Nanes Sarfati et al., 2021). Therefore, we decided to investigate whether the differences in development, growth, and feeding capacity between parasites cultured in either FBS- or HS-complemented medium were associated with distinct cellular proliferation rates. EdU pulse experiments revealed notably lower cell proliferation in FBS-cultured parasites as early as 2 days post-transformation compared to HS-cultured parasites (Figure 4). It has previously been demonstrated that a group of five well-defined somatic stem cells is the only set of cells that actively proliferate in 2-day schistosomula (Wang et al., 2018). Hence, the proliferating cells observed and quantified in our study were most likely stem cells (Figure 4—figure supplement 1; Videos 1 and 2). The difference between the number of proliferating stem cells in parasites cultured in FBS or HS increased significantly over time. HS-cultured schistosomula showed higher numbers of proliferating stem cells, with a median of >48 and>60 EdU + cells per worm at days 8 and 15, respectively (Figure 4). On the other hand, most FBS-cultured parasites displayed no more than an average of 20 EdU + cells per worm (Figure 4). hRBCs were added at day 13 post-cercarial transformation to both FBS- and HS-complemented culture media. Worms kept in FBS or HS in the absence of hRBCs were included as controls. Regardless of the serum employed, no significant differences in the numbers of proliferating cells were observed between worms cultured in the presence or absence of hRBCs (Supplementary file 1B and F).

**Figure 4—figure supplement 1.**
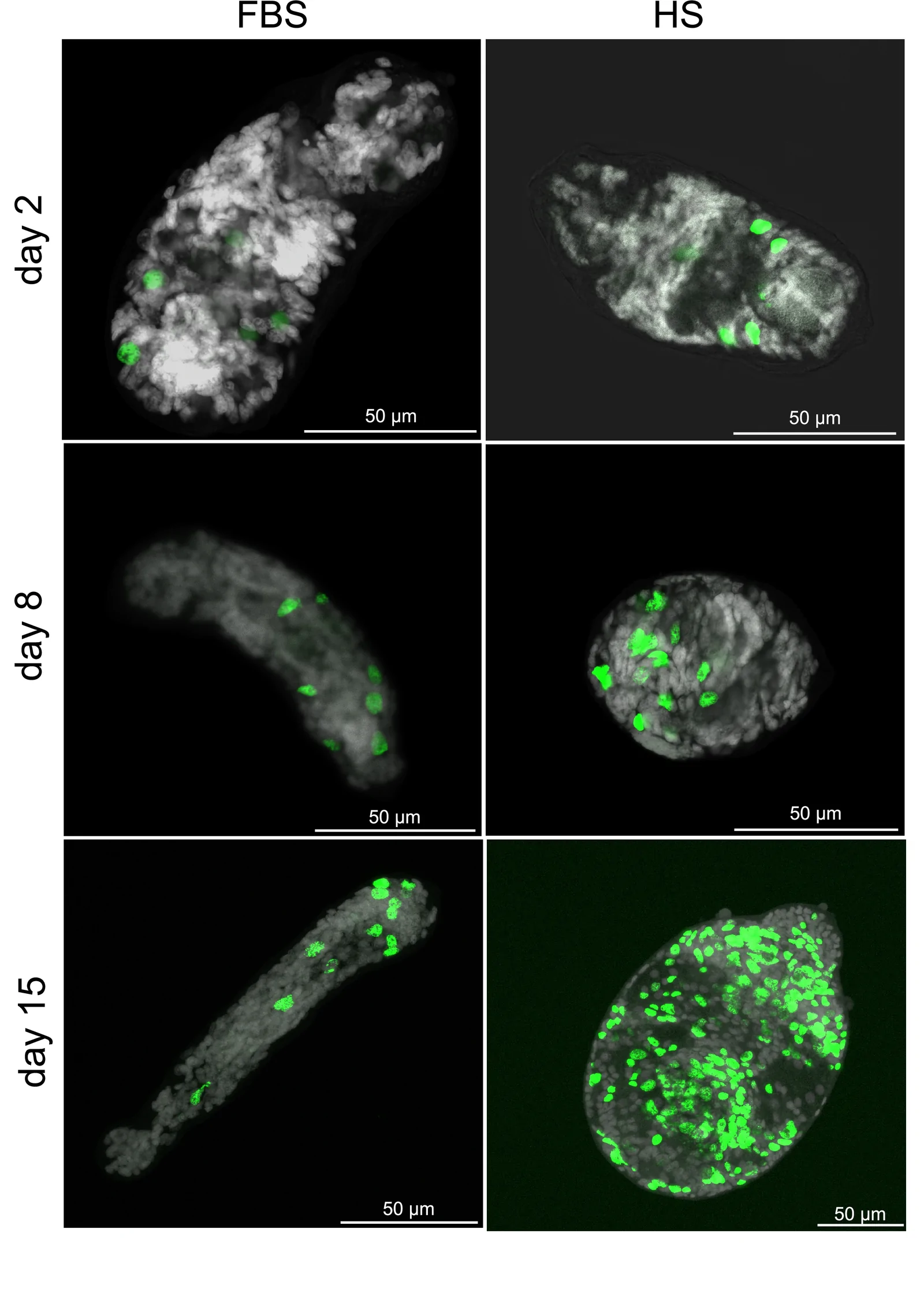
Representative confocal high-magnification images of individual parasites displaying Edu + cells at each indicated time point and culture condition. Edu + cells and nuclei were labelled with Alexa fluor 488 (green) and DAPI (white/grey), respectively. Scale bars: 50 µm.

**Video 1.** Representative Z-stack of EdU + cells after 2 days of in vitro culture with human serum. EdU + cells and nuclei were labelled with Alexa fluor 488 (green) and DAPI (grey), respectively. Scale bars: 50 µm.

**Video 2.** Representative Z-stack of EdU + cells after 2 days of in vitro culture with foetal bovine serum. EdU + cells and nuclei were labelled with Alexa fluor 488 (green) and DAPI (grey), respectively. Scale bars: 50 µm.

### In vitro cultured schistosomes display sexual dimorphism, developing reproductive systems and pairing capacity

**Figure 5.**
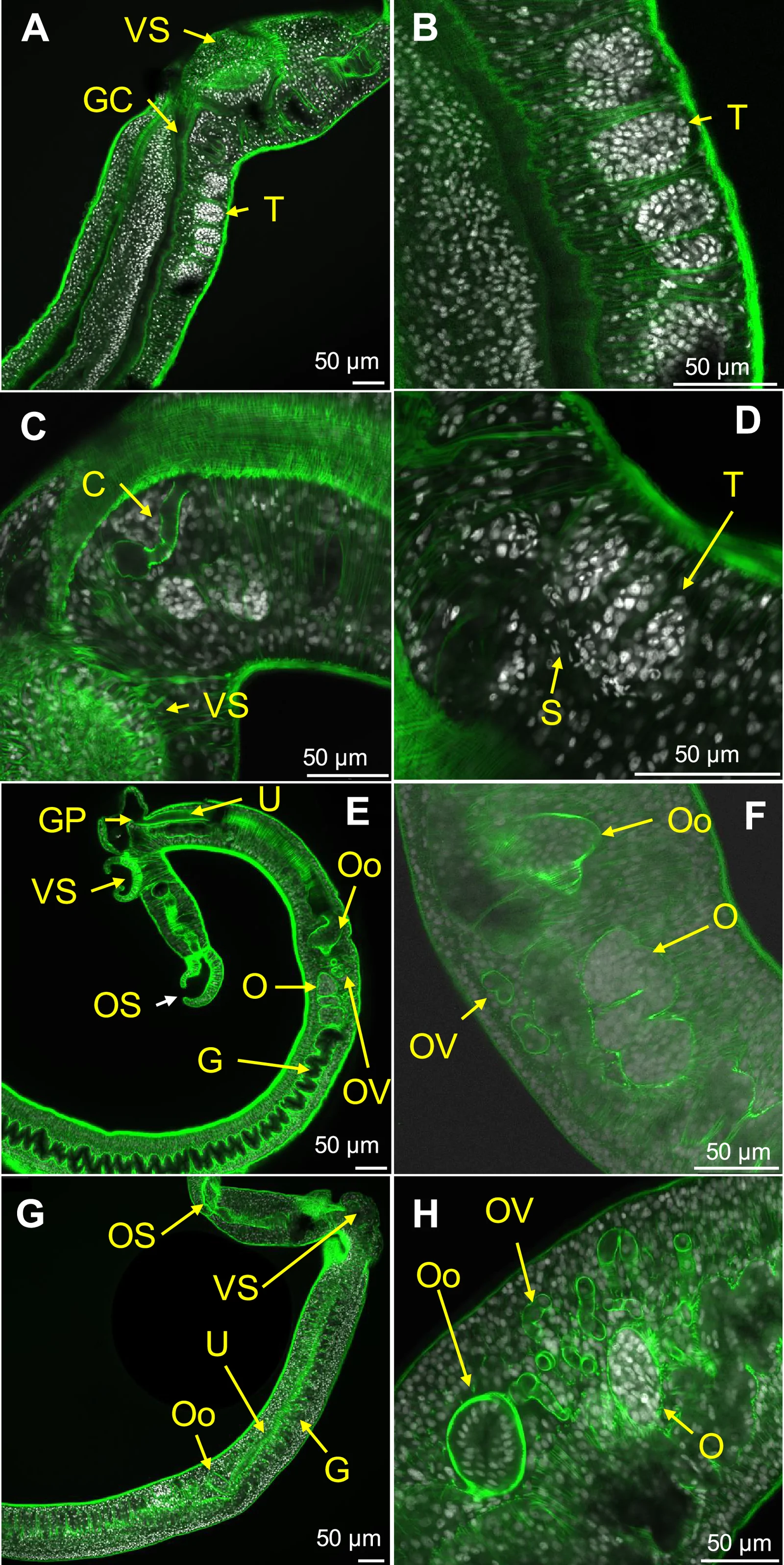
In vitro cultured schistosomes display sexual dimorphism and developing reproductive systems. Representative confocal microscopy images of in vitro developed male (**A–D**) and female (**E–H**) at day 60 in culture. Worms shown in panels G and H are different individuals. CellMask Green Actin Tracking Stain: green, DAPI: grey (**A–H) **. C, cirrus; T, testis; OS, oral sucker; VS, ventral sucker; GC, gynaecophoric canal; G, gut; GP, genital pore; U, uterus; O, ovary; Oo, ootype; OV, oviduc; S, sperm. Scale bar: 50 µm.

**Figure 6.**
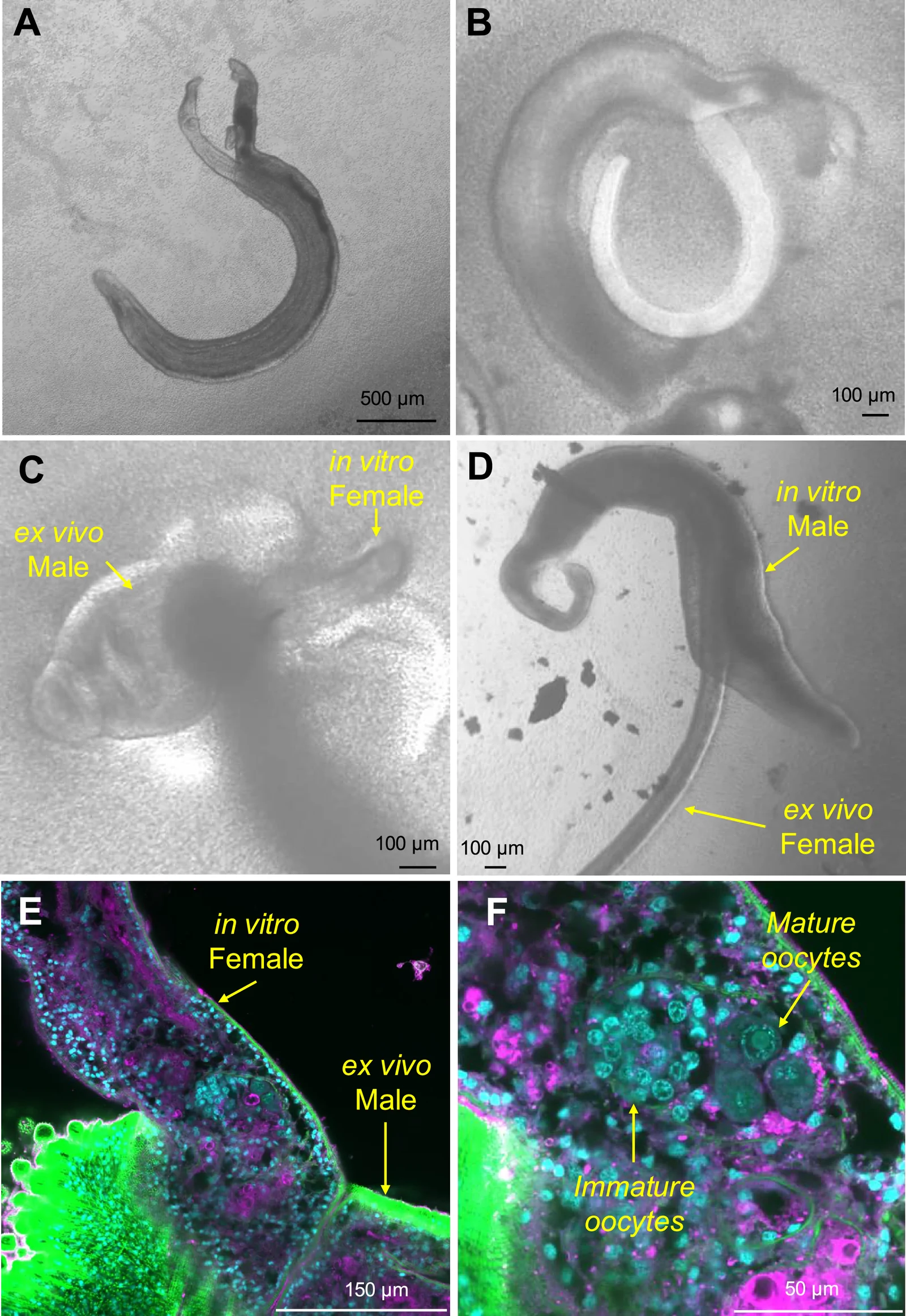
In vitro cultured schistosomes are capable of pairing. (**A, B**) Representative bright-field images of pairs of schistosomes developed entirely in vitro after 80 (**A**) and 150 (**B**) days of culture in culture medium supplemented with HS. Scale bars: 500 µm (**A**), 100 µm (**B**). (**C, D**) Representative bright-field images of a worm pairs between ex vivo-collected males and in vitro-developed females (**C**) and ex vivo-collected females and in vitro-developed males (**D**) within 24 hours after placing the worms in the same well to facilitate pairing. Scale bars: 100 µm, (**E, F**) Confocal microscopy image of a schistosome pair (ex vivo-collected male and in vitro-developed female) in copula (**E**), and magnification of the ovarian area, highlighting maturing oocytes (**F**). CellMask Green Actin Tracking Stain: green, DAPI: cyan , CellMask Deep Red Plasma Membrane Stain: magenta. Scale bar: 150 µm (**E**), 50 µm (**F**).

In vitro-developed schistosomes began to show sexually dimorphic features from day ~42 onwards in HS-supplemented culture medium (Figure 1, week 6). Confocal microscopy of DAPI- and Phalloidin-stained in vitro-cultured male worms confirmed the presence of the gynaecophoric canal, developing three to five testis lobes with sperm cells and cirrus (Figure 5A–D; Video 3). Likewise, we confirmed the presence of primordial ovaries, oviduct, ootype, and uterus in female parasites entirely developed in vitro (Figure 5E–H; Video 4). In some experiments, sexually dimorphic parasites cultured in HS medium were kept alive for more than 150 days (Figure 5—figure supplement 1). While the establishment of sexual dimorphism was robust and reproducible in more than 15 independent experiments, pairing between male and female parasites was rare. Pairing was observed only in experiments lasting more than 80 days in which we were only able to observe a few couples pairing up, and in addition, this pairing was temporary (Figure 6A and B; Video 5).

**Figure 5—figure supplement 1.**
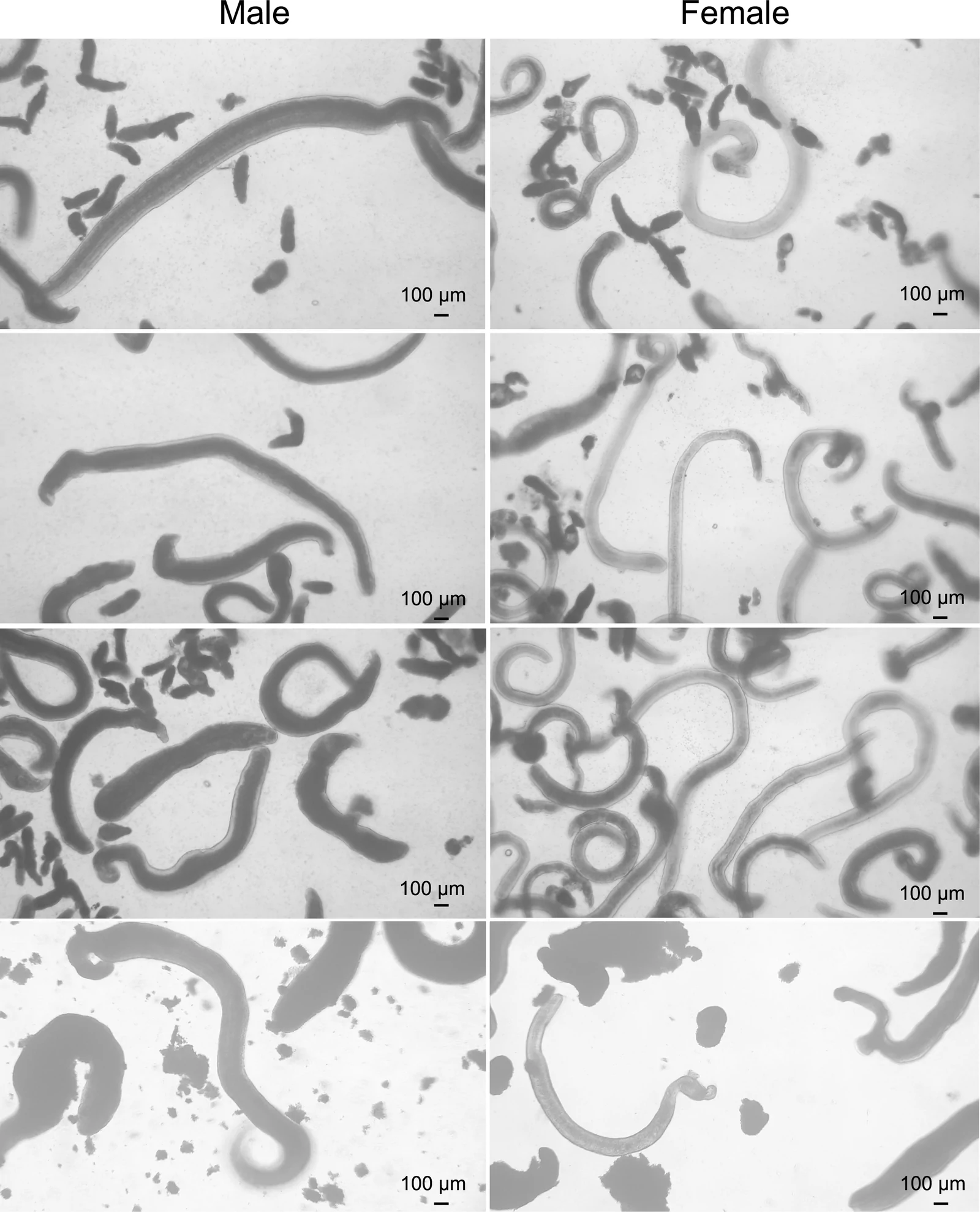
Representative bright-light pictures parasites cultured in HS supplemented medium for more than 100 days, indicating male and female worms. (**A-F**) Parasites cultured for 103 days. (**G, H**) Parasites cultured for 145 days. Scale bar: 100 µm.

**Video 3.** Representative Z-stack of an in vitro developed male worm. CellMask Green Actin Tracking Stain (green), and DAPI-stained nuclei (cyan). Scale bar: 25 µm.

**Video 4.** Representative Z-stack of an in vitro developed female worm. CellMask Green Actin Tracking Stain (green) and DAPI-stained nuclei (cyan). Scale bar: 10 µm.

**Video 5.** Representative video of in vitro developed females and males *in copula* at ~80 days in culture.

Considering the rarity of the pairing between in vitro developed male and female worms, we investigated whether these parasites display the capacity of pairing with in vivo developed worms collected from experimentally infected mice. Male and female adult worms were recovered from mice by portal perfusion on day 42 post-infection, sorted by sex and placed in culture with worms of the opposite sex developed in vitro. Within 24 hours of initiating the co-culturing of in vitro developed worms with ex vivo collected worms, couples were observed (Figure 6C and D; Videos 6 and 7). These findings suggested that the in vitro developed and sexually dimorphic parasites were capable of intersexual pairing. Moreover, in vitro developed females coupled with ex vivo collected mature males displayed signs of primordial ovary maturation with larger oocytes towards the posterior region of the ovary (Figure 6E and F; Videos 8 and 9). On the other hand, females developed in vitro but not paired with ex vivo males remained immature. Remarkably, in more than 30 independent in vitro culture experiments, where male and female parasites developed sexual dimorphism and eventually paired up, no eggs were produced or laid. This indicates that further refinements in the culture protocol are needed to advance parasite development (the in vitro development was delayed compared to the in vivo development), increase the likelihood of pairing and facilitate the production of eggs.

**Video 6.** Representative video of in vivo developed male and in vitro developed female *in copula*.

**Video 7.** Representative video of in vivo developed female and in vitro developed male *in copula*.

**Video 8.** Z-stack of a schistosome pair (in vivo-developed male and in vitro-developed female) *in copula*. CellMask Green Actin Tracking Stain: green, DAPI: grey, CellMask Deep Red Plasma Membrane Stain purple. Scale bar: 25 µm.

**Video 9.** Same Z-stack of a schistosome pair as Video 8 (in vivo-developed male and in vitro-developed female) *in copula* with higher magnification of the ovarian area, highlighting maturing oocytes. CellMask Green Actin Tracking Stain: green, DAPI: grey, CellMask Deep Red Plasma Membrane Stain purple. Scale bars: 50 µm.

## Discussion

Recent progress in culture systems and functional genomic tools is allowing researchers to address long standing questions in helminth biology that were previously inaccessible to experimentation (Arunsan et al., 2019; Kalinna et al., 2024; Quinzo et al., 2022; Stephens et al., 2025; Xie et al., 2025). Among these enduring questions stand those related to the unique sexual biology of the trematode family Schistosomatidae. Schistosomes are dioecious with genetically determined female and male individuals, which is unusual for flatworms (Loker and Brant, 2006; Loker et al., 2025). However, the sexual dimorphism of male and female schistosome worms only becomes established within the mammalian host and represents a critical step towards worm maturation, intersexual pairing, and egg production (Moné and Boissier, 2004). A better understanding of the molecular and cellular basis underlying sexual dimorphism establishment in schistosomes would lead to approaches to block parasite development and life cycle propagation. Studying the sexual development of schistosomes by performing controlled experiments in which parasites can be co-cultured, genetically manipulated or treated with different compounds requires robust and reproducible protocols for long-term in vitro culture.

Culture protocols have recently been developed to maintain ex vivo schistosomes collected from infected mice (Wang et al., 2019; You et al., 2024), or to obtain juvenile parasites from cercariae for drug screening experiments (Frahm et al., 2021; Buchter et al., 2021; Maharjan et al., 2021; Reimers et al., 2015). However, no LTC conditions have been successfully refined to experimentally assess sexual differentiation and dimorphism establishment in schistosomes. Here, aiming to refine a culture medium formulation that supports in vitro parasite development and establishment of sexual dimorphism, we compared the effect of modified Basch’s media (Mann et al., 2010), supplemented with hRBCs and 20% of either FBS or HS. While initial parasite survival and development appeared comparable in both conditions during the first week in culture, striking morphological differences emerged from week 2 onwards. Parasites cultured in HS progressed through all developmental categories and acquired sexual dimorphism by week 6. On the other hand, parasites maintained in FBS were stunted at early stages (mainly lung stage). These experimental outcomes were consistent with the findings reported by Paul F. Basch (Basch, 1981b) in 1980. Probably, from the 1980s onwards a combination of factors that include ethical concerns, variability associated with human-derived products and safety considerations related to blood borne viruses such as HIV and Hepatitis B and C determined that parasitologists favoured the use of FBS over HS for helminth culture. FBS has been extensively used in cell culture media, refined and adapted to diverse human and animal cell types since 1958 (Puck et al., 1958) providing a reliable source of amino acids, carbohydrates, hormones, lipids, proteins, vitamins, and growth factors (van der Valk et al., 2018). Nevertheless, the scientific community is increasingly advocating for the replacement of FBS as a supplement in tissue culture (Gstraunthaler et al., 2013). This trend is driven by limitations associated with FBS, including the presence of undefined tentatively harmful factors for the cells in culture, lack of reproducibility, lack of transparency in its production and critically ethical concerns related to animal welfare (Weber et al., 2025). Our protocol that supports the full in vitro development of schistosomes will also positively impact the 3Rs (Louis-Maerten et al., 2024) by minimising the use of animals for research and serum production (Rosolowski et al., 2025; Subbiahanadar Chelladurai et al., 2021).

Parasites cultured in the presence of HS not only developed into sexually dimorphic male and female worms but strikingly were able to digest hRBCs and process haemoglobin within 1 day after the addition of the cells. These findings may reflect differences in gastrodermis and oesophageal gland development; worms cultured in the presence of HS displayed a differentiated gastrodermis that enabled haemoglobin digestion and accumulation of hemozoin within the intestines (Xiao and Sun, 2017). In contrast, most parasites cultured in FBS were unable to produce hemozoin in the presence of hRBCs. This may be due to impaired gastrodermis differentiation that ultimately would lead to stunted development and death. Haematophagous parasites including *Plasmodium* species and schistosomes obtain key nutrients via the proteolysis of host haemoglobin. However, this process leads to the production of free-haem groups which in turn generate highly toxic oxygen free radicals and lipid peroxidation (Sun et al., 2025). These toxic free-haem derivatives become inactive when aggregated into an inert crystalline polymer, named hemozoin, observed as dark pigment within the intestines of schistosome intra-mammalian stages (Xiao and Sun, 2017). In addition, increasing evidence shows that hemozoin may play critical roles during parasite development by supplying iron for egg production (Xiao and Sun, 2017) and interaction with the mammalian host by an immune modulatory role (Truscott et al., 2013). In the mouse model, schistosomula begin to feed on blood once they have left the lungs and reached the portal system 9–11 days post-infection (Nation et al., 2020; Miller and Wilson, 1980). Moreover, schistosomula collected from experimentally infected mice at day 13 post-infection already show hemozoin in their guts (Wangwiwatsin et al., 2020). Similarly, HS-developed schistosomula were able to feed on blood in vitro within a comparable time window; hRBCs were added in the medium on day 13 in culture, and within 24 hr, most of the parasites’ gut contained hemozoin. These findings suggest that our in vitro culture system successfully recapitulates in vivo gastrodermis development of schistosomula. A better understanding of the in vitro development of the parasite oesophageal gland and gastrodermis and its role in the production of hemozoin will expose tentative novel targets for control (Corrêa Soares et al., 2009).

Although previous reports have shown that it is possible to cultivate parasites in cell-free environments (Maharjan et al., 2021; Frahm et al., 2019), adding hRBC has proven essential for long-term maintenance of parasites and for establishing sexual dimorphism. EdU pulse experiments suggested that schistosome in vitro development in the presence of HS may be driven by the proliferation and differentiation of stem cells. The stem cell system in schistosomes has been extensively studied since the first description of somatic stem cells in adult worms (Collins III et al., 2013), followed by single-cell transcriptomic identification and functional characterisation of three key stem cell populations in intra-snail stages (Wang et al., 2018). Two of these stem cell types, maintained in intra-mammalian stages, proliferate and differentiate into precursors of somatic and germ line cells throughout development (Li et al., 2021). Although we have not performed transcriptomic analyses in this study, the positive correlation between number of EdU+ cells and growth (determined by increasing worm areas) and gross development in HS-developed parasites was evident already within the first 48 hr in culture. These findings indicate that stem cells may play a central role in driving organised growth, tissue differentiation, and ultimately the establishment of the sexual dimorphism in HS-developed parasites. Conversely, FBS-cultured parasites displayed no more than 20 EdU + cells per worm on average, reaching a plateau in the number of proliferating cells from day 8 in culture, which probably underlie the arrested development of these worms. Overall, the FBS-cultured parasites did not progress beyond the lung stage. Consistent with these findings, confocal microscopy imaging revealed in HS-developed parasites clear somatic and reproductive anatomical structures, including male gynaecophoric canals and testes with sperm cells, as well as female ovary primordia, oviducts, ootypes, and uteri. These developmental features illustrate the capacity of our culture system to capture key biological transitions previously only accessible from experimentally infected animal models. That said, while our system was highly efficient in producing sexually dimorphic worms, spontaneous pairing between male and female parasites was extremely rare, mainly in aged in vitro cultures (from 80 to 100 days in culture) indicating that other factors, for example cholesterol, may be missing (Wang et al., 2019). In any case, we decided to test the pairing capacity of the dimorphic worms by conducting pairing experiments between in vitro-developed females and ex vivo-collected males, and vice versa. Strikingly, couples were observed within 24 hr after co-cultivating in vivo and ex vivo opposite sex parasites, albeit no eggs were produced. However, we observed evidence of pairing-trigger oocyte maturation in in vitro-developed females paired with ex vivo-collected male worms. Our research group has recently been involved in the discovery and functional characterisation of a transcription factor of the retinoic acid receptor family, SmRAR, and related genes (Moescheid et al., 2025). SmRAR and associated genes may play a key role in oocyte differentiation triggered by pairing. Our pairing experiments and confocal imaging analyses suggested that in vitro developed female oocytes started to differentiate after pairing with an in vivo developed male worm, probably mediated by the activation of the SmRAR pathway (Moescheid et al., 2025). Moreover, the involvement of a male-derived nonribosomal peptide pheromone, that is β-alanyl-tryptamine or ‘BATT’, in the pairing-driven female sexual maturation was demonstrated (Chen et al., 2022). In addition, soluble factors produced by the worms with effect on the opposite sex cannot be ruled out (Shakir et al., 2023). It has been suggested that host-derived factors, such as the cytokine transforming growth factor β (TGF-β) may be critical for female reproductive development and embryogenesis (Doenhoff et al., 2019). More recently, other host-derived factors that include cholesterol and ascorbic acid have been shown to be critical for maintaining ex vivo fully mature females collected from infected mice (Wang et al., 2019). Considering these elements in future experiments will help overcome the limitations encountered in this study, including the low rate of spontaneous pairing between in vitro-developed male and female worms and the requirement for extended culture periods (>70 days). In addition, further research is needed to assess the role of host- and parasite-derived cues in schistosome development (Walker et al., 2025).

In summary, we have demonstrated that the presence of HS is essential to support fully in vitro development of *S. mansoni* parasites from mechanically transformed cercariae to sexually dimorphic adults. Differences in the numbers of proliferating stem cells between worms developed in HS versus FBS were observed as early as 48 hr in culture. These findings may raise some concerns about studies that rely on in vitro culture protocols using FBS, including those focused on parasite developmental biology, interaction with the host and drug screening. HS contains essential host-specific molecules that are absent or insufficient in FBS, and future efforts should aim to identify these factors. Optimising schistosome LTC systems will: (1) deepen our understanding of fundamental aspects of schistosome biology, development and host–parasite molecular crosstalk; (2) positively impact the 3Rs principles for animal research; and (3) reveal tentative targets for novel control strategies.

## Materials and methods

**Key resources table:** 

Reagent type (species) or resource	Designation	Source or reference	Identifiers	Additional information
Biological sample (*S. mansoni*)	*S. mansoni* NMRI strain	Wellcome Sanger Institute (WSI) and Aberystwyth University (AU)		
Biological sample (*Biomphalaria glabrata*)	*Bi. glabrata*	Geyer et al., 2017		
Sequence-based reagent	W1a – forward	Grevelding, 1999	PCR primers	5′-CAACACAGTGAAATTCTTCC-3′
Sequence-based reagent	W1b – reverse	Grevelding, 1999	PCR primers	5′-GAATTCACCACTCGACATTC-3′
Sequence-based reagent	Actin – forward	Rinaldi et al., 2009	PCR primers	5′-CAG TGT TCC CTT CCA TCG TT-3′
Sequence-based reagent	Actin – reverse	Rinaldi et al., 2009	PCR primers	5′-GGA CAG GGT GTT CTT CTG GA-3′
Chemical compound, drug	5-Ethynyl-2'-deoxyuridine (EdU)	Cambridge bioscience	61135-33-9	
Chemical compound, drug	Azide fluor 488	Sigma-Aldrich	760765–1 MG	
Chemical compound, drug	Fluoromount-G Mounting Medium, with DAPI	Invitrogen	00-4959-52	
Chemical compound, drug	CellMaskTM Green Actin Tracking Stain	Invitrogen	17163269	
Chemical compound, drug	CellMaskTM Deep Red Plasma Membrane Stain	Invitrogen	C10046	
Chemical compound, drug	NucBlue Live ReadyProbes Reagent	Invitrogen	R37606	
Chemical compound, drug	Lactalbumin hydrolysate	Merk Life Sciences	Cat. 61300–500 G	
Chemical compound, drug	Hypoxanthine	Merk Life Sciences	Cat. H9636-1G	
Chemical compound, drug	Serotonin	Merk Life Sciences	Cat. H9523	
Chemical compound, drug	Hydrocortisone	Merk Life Sciences	Cat. H0888-1G	
Chemical compound, drug	Triiodothyronine	Merk Life Sciences	Cat. T6397-100 mg	
Chemical compound, drug	MEM vitamins	Merk Life Sciences	Cat. M6895-100 ml	
Chemical compound, drug	Schneider’s insect media	Merk Life Sciences	Cat. 50146–500 ml	
Chemical compound, drug	Hepes	Merk Life Sciences	Cat. H0887-100 ml	
Chemical compound, drug	Heat-inactivated human serum	NHSBT serum	Cat. NC02	
Chemical compound, drug	Heat foetal bovine serum	Thermo Fisher Scientific	Cat. 11550356	
Chemical compound, drug	Antibiotic antimycotic solution	Thermo Fisher Scientific	Cat. 15140–122	
Chemical compound, drug	Insulin solution	Merk Life Sciences	Cat. I9278-5 ml	
Chemical compound, drug	L-glutamine	Thermo Fisher Scientific	Cat. 11539876	
Chemical compound, drug	Human red blood cells	NHSBT-NCI	Cat. NC15	
Software, algorithm	CVAT (Computer Vision Annotation Tool) online server	https://www.cvat.ai/		

### Cercariae transformation and culture of schistosomula

*S. mansoni* (NMRI strain) schistosomula were obtained and cultured as previously described with minor modifications (Mann et al., 2010). In brief, patent mix strain of *B. glabrata* snails (Geyer et al., 2017) experimentally infected *en masse* with ~20 miracidia per snail, were thoroughly rinsed, transferred to Lepple water (~100 ml) and exposed to light for 2 hr at 26 °C to induce cercarial shedding. Cercariae were collected, passed through a 100 μm filter into 50 ml tubes to remove snail debris and faeces, placed 1 hy on ice, and concentrated by centrifugation (300 × *g* for 3 min at 4 °C with half break deceleration). Cercariae were washed three times in 1 X PBS supplemented with 200 U/ml penicillin, 200 μg/ml streptomycin, and 500 ng/ml amphotericin B (Merck). During the washes, the cercarial pellets were successively combined and finally resuspended in ‘Schistosomula wash medium’ (Dulbecco’s modified Eagle medium – DMEM, supplemented with 10 mM Hepes, 200 U/ml penicillin, 200 g/ml streptomycin, and 500 ng/ml amphotericin B). Cercariae were vortexed full speed for 30 s and placed on ice for 1 min. Thereafter, the cercarial tails were sheared off by ~10 back and forth passes through a 19-gauge (19 G) needle, an ~3 μl aliquot of parasites inspected under microscope to confirm that >90% of cercariae had their tail removed, and the cercarial heads were separated from the tails by a Percoll (Merck) gradient (1.5:1 percoll:DMEM) and centrifugation (300 × *g* for 15 min at 4 °C, half break deceleration). The pellet containing purified cercarial heads, was collected and washed three times by centrifugation (300 × *g* for 3 min at 4 °C) in ‘Schistosomula wash medium’. Newly transformed schistosomula were counted in twelve 5-μl aliquots under the microscope. To reduce parasite mortality due to a high density of worms, ~8000 schistosomula were transferred to each well of a six-well tissue culture plate (Thermo Fisher Scientific, Loughborough, UK) containing 5 ml of modified Basch’s medium supplemented with additives and FBS or HS heat-inactivated FBS or HS serum, that is ‘LTC medium’ as indicated in Table 1. The number of parasites cultured per well (~8000 schistosomula) was determined empirically, as no formal titration experiments were performed. At higher densities (>10,000 per well), more frequent media changes were required, and parasite development appeared to be impaired. Parasites were cultured in a tissue culture incubator at 37 °C under 5% CO_2_ in air. LTC medium was replaced twice a week and washed hRBCs added to a final concentration of 0.02% v/v at day 13 after transformation. Washed hRBCs were replaced every 2 weeks, or sooner if their numbers decreased due to consumption.

**Table 1.** Long-term culture medium (LTC medium) composition.

Reagent name	Final concentration	Reagent source
DMEM, high glucose, sodium pyruvate		Thermo Fisher Scientific (Cat. 13476146)
Lactalbumin hydrolysate	1 mg/ml	Merk Life Sciences (Cat. 61300–500 G)
Hypoxanthine	500 nM	Merk Life Sciences (Cat. H9636-1G)
Serotonin	1 μM	Merk Life Sciences (Cat. H9523)
Hydrocortisone	1 μM	Merk Life Sciences (Cat. H0888-1G)
Triiodothyronine	200 nM	Merk Life Sciences (Cat. T6397-100MG)
MEM vitamins	1X	Merk Life Sciences (Cat. M6895-100mL)
Schneider’s insect media	10%	Merk Life Sciences (Cat. 50146–500 mL)
Hepes	20 mM	Merk Life Sciences (Cat. H0887-100mL)
Heat-inactivated human serum Foetal bovine serum	20%	HS: NHSBT serum (Cat. NC02) FBS: Fisher Scientific (Cat. 11550356)
Antibiotic antimycotic solution	2X	Thermo Fisher Scientific (Cat. 15140–122)
Insulin solution	8 μg/ml	Merk Life Sciences (Cat. I9278-5mL)
L-glutamine	2 mM	Thermo Fisher Scientific (Cat. 11539876)
Human red blood cells	0.02% v/v	NHSBT-NCI (Cat. NC15)

All human blood products, obtained from NHSBT-NCI, were handled with universal precautions within a biosafety cabinet class II and suitable PPE. The HS was heat-inactivated at 56 °C for 30 min in an incubator, aliquoted, and stored at –20 °C until use. The hRBC were washed in ‘Schistosomula wash medium’ and stored at 4 °C until use as previously described (Mann et al., 2010). Briefly, the hRBCs were transferred from the pack to 50 ml tubes and washed by centrifugation at 500 × *g* for 5 min at 4 °C. Half the supernatant was removed, tubes’ content combined, centrifuged as above and resuspended in ~25 ml of ‘Schistosomula wash medium’. The washes were repeated four more times.

### Phenotype scoring of developing schistosomula

To analyse the in vitro development of schistosomula, images and videos were taken at regular intervals of at least once a week in >10 independent culture experiments, using a digital Euromex camera connected to an Olympus CK2 optical microscope. Up to 18 pictures per experiment with an average of ~400 worms per picture were used for parasite staging from at least five independent experiments (Supplementary file 1A). The investigators who assigned categories were blinded to experimental conditions. Based on a well-defined staging system (Wangwiwatsin et al., 2020; Basch, 1981a), the parasites were classified into six developmental categories as described (Maharjan et al., 2021; Figure 1—figure supplement 1); category 0: dead parasites showing granulation, rounded shape and degraded or broken tegument – the number of dead parasites may be underestimated due to the loss of some dead worms during the media change; category 1: newly transformed schistosomula, corresponding to the in vivo ‘skin stage’, these worms retain the cercarial head shape with no visible internal structures such as gut; category 2: lung stage schistosomula, elongated and slim worms, usually with a bulged end; category 3: early liver stage, bigger and wider worms compared to the previous stage, with hemozoin pigment (i.e. haemoglobin degradation product) and two caeca gut; category 4: late liver stage, in which the gut is further developed, the two intestinal caeca have fused behind the ventral sucker, and the parasites started to acquire an evident vermiform shape; category 5: sexually dimorphic stage, comprising large, vermiform male- and female-looking worms. The females are longer and thinner than males, the caeca fusion localises closer to the anterior end of the worm (i.e. ~⅓ of the worm length from the anterior end), and both suckers are smaller compared to the male suckers. The male worms are characterised by a larger and wider head, larger suckers, the caeca fusion closer to the posterior end of the animal (i.e. ~⅔ of the worm length from the anterior end), and a clearly visible gynaecophoral groove (Wangwiwatsin et al., 2020; Basch, 1981a). To evaluate differences in mortality between HS- and FBS-cultured parasites, data from five experiments were combined and analysed using a Shapiro-Wilk normality test to test normality of the data and a non-parametric Wilcoxon rank sum exact test (Supplementary file 1A and F).

To quantify positive or negative parasites for the presence of hemozoin within their intestines, that is BG+ or BG- parasites, respectively, hRBCs were added to the culture at day 13, and 1 or 2 days later bright field microscopy images were taken. Counts of BG +and BG- parasites were collected from five independent in vitro culture experiments (Supplementary file 1D). Statistical analysis was performed using a Wilcoxon rank-sum (Mann-Whitney) test, with p≤0.05 considered significant (Supplementary file 1F).

### Measurement of parasite area

Images of in vitro developing parasites were taken at weeks 1, 2, 3, 4, 6, 8, and 10 after cercariae transformation with an Euromex camera fitted to an Olympus CK2 optical microscope at ×4 and ×10 magnification. For these magnifications, images of scale bars were captured for pixel to area values (µm²). Parasites from five independent LTC experiments were masked in COCO format from captured images using the CVAT (Computer Vision Annotation Tool) online server (https://www.cvat.ai/). Area metrics were then calculated for each parasite by leveraging the pycocotools package in a customised Python script (DOI 10.5281/zenodo.21992371). Raw and processed data is provided in Supplementary file 1C. Normal distribution was checked using Shapiro test and Mann Whitney tests performed with a significant value set up for p≤0.05 (Supplementary file 1F).

### PCR for sexing and confirmation of sexual dimorphism

To confirm the in vitro establishment of sexual dimorphism, parasites were individually collected from culture by day ~80, for blind sex assignment by bright-field microscopy and confirmation by sex-specific PCR (Buddenborg et al., 2023; Figure 1—figure supplement 3). Briefly, pictures from individual in vitro developed parasites were taken and sex assigned based on morphological features. Thereafter, genomic DNA from the sex-assigned individual parasites was extracted by lysing the worms at 95 °C for 1 hr in 50 mM NaOH, 0.4 mM disodium ethylenediaminetetraacetic acid (EDTA), followed by the addition of neutralising buffer (80 mM Tris–HCl in water). Samples were stored at 4 °C until use. PCR reactions were performed in a final volume of 10 μl consisting of 5 μl Platinum 2 X hot start PCR master mix (Invitrogen, California, USA), 0.5 μl (10 μM stock) of forward and reverse primers targeting W1 repeat (Grevelding, 1999) and actin (Rinaldi et al., 2009 Supplementary file 1B), 1 μl of template DNA and 3 μl of water. The PCR program comprised an initial denature step at 94 °C for 2 min followed by 30 cycles of denature at 98 °C for 5 s, and annealing/elongation at 60 °C for 15 s. The PCR products were resolved on a 1% agarose gel using 1% TAE buffer and 1 kb GeneRuler DNA ladder (Thermo Fisher Scientific, UK).

### Detection and quantification of proliferating cells

Parasites cultured for 15 days (D15) in media supplemented with either HS or FBS and hRBC added at D13 were harvested at days 2, 8, or 15 after cercariae transformation. Parasites cultured under the same conditions but with no hRBC were included as controls. The day before each indicated time point, a solution of 5-ethynyl-2'-deoxyuridine (EdU) was added to the culture medium at 10 μM final concentration. At each indicated time point, parasites from each group were collected and transferred to a 35 µm mesh basket (CEM Microwave Tech) in a well of a 24-well plate, washed in 1 x PBS, fixed in 4% paraformaldehyde (PFA) in 1 x PBS for 30 min, washed in 1 x PBS and stored at 4 °C until use. The worms were incubated in PBST (1 x PBS +0.3% Triton x-100) containing 2 μl of Proteinase K (20 mg/ml) for 5 min at room temperature, washed three times in PBST, fixed in 4% PFA in 1 x PBS for 10 min at room temperature and washed five times in PBST. EdU-positive cells were revealed by incubating the parasites for 30 min in the dark in a solution containing Azide fluor 488 (Sigma-Aldrich), diluted in a solution of 1 mM CuSo4, 0.025 mM Azide fluor 488, 95 mM L-ascorbic acid in 1 x PBS. The worms were washed five times in PBST and incubated in mounting media containing DAPI (Fluoromount-G Mounting Medium, with DAPI, Invitrogen) before mounting for confocal microscopy (Leica SP8 super resolution laser confocal microscope). EdU + cells per parasite were counted for an average of 100 parasites across three independent experiments (Supplementary file 1D). Worms were grouped based on the number of cells per individual, but all those showing ⪰60 EdU + cells were counted in the same group named ‘60 EdU + cells’. Therefore, the data were considered ordinal and the statistical analysis performed by Kruskal-Wallis test with Dunn multiple comparison post-hoc test, with p≤0.05 considered significant (Supplementary file 1F).

### Pairing experiments

*S. mansoni* adult male and female worms were collected from experimentally infected mice at 47 days post infection (Mann et al., 2010). Briefly, mice were euthanised by intraperitoneal injection of 200 μl of 200 mg/ml pentobarbital supplemented with 100 U/ml heparin, and worms collected by portal perfusion (the hepatic portal vein was sectioned followed by intracardiac perfusion with phenol-red-free DMEM, containing 10 U/ml heparin) and washed in DMEM. In 24-well plates, in vitro developed female worms were cultured in the presence of in vivo developed male worms and vice versa at a ratio of one female every two males in the well containing culture medium (Table 1) supplemented with 20% HS. The parasites were cultured for several days in an incubator at 37 °C, 5% CO_2_ and checked daily under microscope for the presence of pairing.

### Parasite staining and confocal imaging

Live parasites collected from culture at indicated time points were stained with 1:1000 dilutions of CellMaskTM Green Actin Tracking Stain (Invitrogen) and CellMaskTM Deep Red Plasma Membrane Stain (Invitrogen) to label polymerised/filamentous actin (F-actin) and cell membranes, respectively. Nuclei were stained by adding two drops per millilitre of NucBlueTM Live ReadyProbesTM Reagent (Invitrogen). After an overnight incubation at 37 °C, worms were collected in 15 ml tubes, washed by gravity three times in 1 x PBS, fixed in 4% PFA (in 1 x PBS) overnight at 4 °C, washed three times in 1 x PBS and stored in 200 μl of Fluoromount-G, with DAPI (Invitrogen) before mounting on slides for confocal microscopy. All the images were acquired using a Leica SP8 super resolution laser confocal microscope.

## Data Availability

All raw data and statistical analyses provided in Supplementary file 1.

## Additional files

Supplementary file 1. Supplementary tables. Table A. Raw counts of parasites within each developmental stage category. Each row corresponds to a picture of parasites in culture medium containing FBS or HS. Each column corresponds to the raw parasite counts at indicated stage development (categories 0–5), time in culture (Time in days - D), and experimental condition. Table B. Primers used in PCR for sexing parasites. Table C. Area raw measurements of developing worms. Table D. Percentage of parasites with either black positive (hemozoin) or black negative (no hemozoin) intestine. BG: Black gut. Table E. Raw counting of EdU positive cells per parasite for indicated experimental group, replicate and experiment in long format. The worms were classified by group (column C) and replicate (column D), using the following code: E (‘early’), M (‘medium’) and L (‘late’), corresponding to days 2, 8 and 15, respectively. R and W correspond to conditions with (R) or without (W) human red blood cells, and HS and FBS to culture medium employed. Table F. Summary of all statistical tests employed in this study. 1. Statistical tests of parasite mortality and the raw data table used for this test. 2. Statistical tests for worm size comparisons (correspond to Figure 2). 3. Statistical tests for worm black gut comparisons (correspond to Figure 3). BG: Black gut. 4. Statistical tests for EdU positive cells comparisons (correspond to Figure 4). Replicate code: E, M and L correspond to day 2, 8 and 15 respectively; R and W correspond to the presence (R) or absence (W) of RBCs added 13 days after transformation.

MDAR checklist
