# Supplementary material for: In vitro sexual dimorphism establishment in schistosomes
Source: eLife. 2026 Sep 10;15:RP111066. doi: 10.7554/eLife.111066 (PMC13561514; doi:10.7554/eLife.111066)
Supplement: Peer review history

# Editor report: eLife Assessment

**DOI:** 10.7554/eLife.111066.3.sa0

**Authors:** Dominique Soldati-Favre

**Affiliations:** University of Geneva Switzerland

**Keywords:** Compelling, Valuable

This **valuable** study establishes an improved long-term in vitro culture system for *Schistosoma mansoni* that enables progression of juvenile parasites to advanced developmental stages exhibiting sexual dimorphism. The work has significant implications for experimental studies of schistosome development and for reducing dependence on animal infection models. The evidence is **compelling**, supported by robust phenotypic characterization, and integrated molecular and metabolic analyses. The results show that host-derived culture conditions promote essential developmental programs associated with parasite maturation, although the system does not fully recapitulate reproductive development, as evidenced by low pairing frequencies and the lack of egg production.

# Referee report: Reviewer #2 (Public review)

**DOI:** 10.7554/eLife.111066.3.sa1

Summary:

The authors perform confirmation studies of Paul Basch's seminal schistosome work from 1981, demonstrating the development of transformed schistosomules into sexually dimorphic adult parasites, albeit without successful egg production. In addition to the findings from Basch's earlier work, the authors add some new molecular data in the form of analysis of proliferative cells in in-vitro derived animals.

Strengths:

The authors successfully confirm experimental results from earlier schistosome researchers, providing a potential new tool for studying schistosome biology without the need for vertebrate hosts.

Weaknesses:

The display of data from the authors is sometimes difficult to follow/understand where it comes from. For example:

(1) Line 136: the authors claim state that parasites in HS and FBS conditions have substantially different mortality rates (11.3 +/- 2.7 vs 5 +/- 2.3) but a quite high p-value (0.8). Analyzing the raw data myself, this reviewer obtained a mean of 8.2 +/- 1.7% vs 4.8% +/- 4.3% with a p-value 0f 0.15. Either the data are not clearly presented, and this reviewer did not follow them, or the data presented in the text do not match the raw data in the supplemental files.

(2) Line 187/Figure 4: though it is not clearly stated, it appears that the authors treat their EdU counts as an ordinal data set of 61 steps (from 0 to >60) rather than a continuous measure of EdU+ cells per animal. In this author's opinion, the graph strongly suggests a continuous data set, and the fact that this reviewer had to dig through poorly-labeled raw data to discover the nature of the data is problematic. The authors should either switch to a continuous data set or make it explicit that the data shown are ordinal. If counting EdU+ cells is too arduous, the authors could consider comparing the amount of EdU+ area to the amount of DAPI+ area in maximum intensity projections of their confocal images, as this would roughly approximate the amount of proliferative cells in the animals.

There are some minor issues as well:

(1) Line 122: it is perhaps incorrect to refer to humans as "the" definitive host of schistosomes, as S. japonicum is primarily considered a zoonotic infection with water buffalo/cows being the primary definitive host.

(2) Line 185/298 the authors refer to EdU pulse-chase experiments, but the experiments described here are EdU pulse experiments.

Comments on revised version.

Following the initial submission of the manuscript and a round of peer review, the authors updated the manuscript and addressed all of this reviewer's concerns. As such, this reviewer believes that the manuscript is substantially clearer and will serve as useful literature in the field of schistosome research.

# Referee report: Reviewer #3 (Public review)

**DOI:** 10.7554/eLife.111066.3.sa2

Summary:

This study is significant as it established a protocol for the long-term culture of Schistosoma mansoni newly transformed cercariae which developed in vitro into sexually dimorphic forms. The impact of two different sera, Fetal Bovine Serum (FBS) and Human Serum (HS), added to the culture medium supplemented with human red blood cells was evaluated. The authors demonstrated that HS-cultured parasites were able to digest red blood cells, a critical step for long term parasite development. Furthermore, while most FBS-cultured parasites did not progress beyond an early liver stage, sexual dimorphism was clearly evident in the HS-cultured worms, albeit delayed compared to in vivo development.

Strengths:

This study could contribute to further in vitro studies for a better understanding of the unique sexual biology of Schistosoma mansoni and for screening novel schistosomicidal compounds. By increasing parasite development in in vitro studies this protocol could have a positive impact on the principles of the 3Rs (Replacement, Reduction and Refinement) for animal research.

Weaknesses:

As the authors mentioned "pairing between male and female parasites was rare. Pairing was rarely observed and only after day ~ 80 in culture. Egg production was also not achieved with this protocol.

Comments on revised version.

Some data presentation has been improved as suggested by other reviewers in the revised manuscript. The authors have also clarified the limitations of their long-term culture protocol for Schistosoma mansoni newly transformed cercariae which develop in vitro into sexually dimorphic forms with regards to male and female pairing. Additionally, they addressed my specific question regarding the culture conditions used for ex vivo/in vitro mating. The experimental conditions tested for in vitro developed parasites were the same as those for the pairing experiments. It remains to be investigated the factors that negatively influence pairing during the long-term in vitro culture of Schistosoma.

# Author comment: Author response

**DOI:** 10.7554/eLife.111066.3.sa3

**Authors:** Remi Pichon, Magda E Lotkowska, Jude LD Bulathsinghalage, Madeleine McMath, Mary Evans, Benjamin J Hulme, Kirsty Ambridge, Geetha Sankaranarayanan, Simon Kershenbaum, Sarah D Davey, Josephine Forde-Thomas, Karl F Hoffmann, Matthew Berriman, Gabriel Rinaldi

**Affiliations:** University of Oxford Oxford United Kingdom; Wellcome Trust Sanger Institute Hinxton United Kingdom; Aberystwyth University Aberystwyth United Kingdom; Aberystwyth University Aberystwyth United Kingdom; Aberystwyth University Aberystwyth United Kingdom; Aberystwyth University Aberystwyth United Kingdom; Wellcome Sanger Institute Hinxton United Kingdom; Wellcome Sanger Institute Hinxton United Kingdom; University of Oxford Oxford United Kingdom; Aberystwyth University Aberystwyth United Kingdom; Aberystwyth University Aberystwyth United Kingdom; Aberystwyth University Aberystwyth United Kingdom; University of Glasgow Glasgow United Kingdom; University of Oxford Oxford United Kingdom

The following is the authors’ response to the original reviews.

**eLife Assessment**

This useful study presents an improved protocol for long-term in vitro culture of Schistosoma mansoni that enables progression toward sexually dimorphic stages, representing a meaningful advance for studying parasite development and reducing reliance on animal models. The findings show that host-specific culture conditions support essential developmental and metabolic functions required for parasite maturation, although development remains delayed compared to in vivo conditions. The evidence is solid overall, but limited pairing efficiency and the absence of egg production indicate that the system does not yet fully recapitulate complete reproductive development.

On behalf of the co-authors, we thank the three reviewers and the editors for their complimentary remarks as well as the major and minor comments/ concerns. Addressing these concerns have led to revisions that improved the manuscript. In particular, further analyses have generated an updated Figures 3 and 4, and Supplementary Tables S1, and S4-S6.

**Public Reviews:**

**Reviewer #1 (Public review):**

Pichon, Rémi et al. describe an in vitro method for transforming Schistosoma cercariae into mature adult worms. The authors show that human serum (HS) supports parasite growth and differentiation more effectively than fetal bovine serum (FBS). They also observed differences in parasite growth and activity, with worms cultured in HS efficiently digesting human red blood cells (hRBC). Cultured worms were able to pair with ex vivo adult worms and produce eggs, indicating functional maturation suitable for downstream applications such as drug screening. While the experimental approach is comprehensive and supports the advantage of HS culture conditions, the pairing efficiency was low (≈7%) and required long culture periods (70-80 days), highlighting limitations that may affect reproducibility.

We acknowledge the reviewer for the positive highlights. Regarding the low in vitro pairing efficiency, we have now edited the manuscript to clarify a misleading statement related to 7%. We decided to remove the value of 7% — which corresponds to the percentage of experiments in which couples were observed, as it does not accurately represent the actual number of observed worm pairs and it is probably misleading. We have updated the text as follows:

Results, lines 230 ff.:

“While the establishment of sexual dimorphism was robust and reproducible across more than 15 independent experiments, pairing between male and female parasites was rare. Pairing was observed only in experiments lasting more than 80 days in which we were only able to observe a few couples. In addition, these pairings were temporary (Figures 6A, B; Supplementary Video S4).”

We also agree with the reviewer that the extended culture periods required to obtain fully sexually dimorphic parasites remain a limitation. As elaborated in Discussion (see below), key factors, probably derived from the host, are missing in the in vitro system explaining both the slow in vitro development and low rate of spontaneous pairing between in vitro developed, sexually dimorphic male and female worms. This was discussed as follows (lines 340-343): “That said, while our system was highly efficient in producing sexually dimorphic worms, spontaneous pairing between male and female parasites was extremely rare, mainly in aged in vitro cultures (from 80 to 100 days in culture) indicating that other factors, e.g., cholesterol, may be missing [35].”

A major strength of the study, in particular, is that the authors clearly differentiate the effects of FBS versus HS on developmental progression. The conversion rate observed in HS cultures is significant and consistent with previously published data.

While the study has several strengths, some aspects of the work are not fully explored. In particular, the role of hRBC supplementation requires further clarification. Although HScultured worms were shown to digest hRBC more readily, the implications of this observation remain unclear. Specifically, it would be useful to understand whether hRBC supplementation influences (1) long-term culture stability, (2) molecular pathways associated with development and differentiation, or (3) the pairing capacity of the worms. While addressing these questions may not be the main objective of the study, further discussion of these points would strengthen the manuscript.

We agree that deciphering the role of the human Red Blood Cells (hRBCs) supplementation is critical. Regarding the influence of hRBCs on the long-term culture stability in parasite development it has been well established for more than four decades that schistosomes do need red blood cells to grow in culture [Basch, P. F. Cultivation of Schistosoma mansoni in vitro. II. production of infertile eggs by worm pairs cultured from cercariae. J Parasitol 67, 186-190 (1981); Basch, P. F. Cultivation of Schistosoma mansoni in vitro. I. Establishment of cultures from cercariae and development until pairing. J. Parasitol. 67, 179-185 (1981)]. The molecular pathways underlying development, sexual differentiation and pairing and modulated by hRBCs in culture is currently being investigated by our team. We decided not to include these data and analyses in the current manuscript, as they fall outside its scope.

The manuscript is clearly written and represents a valuable contribution to the field. Overall, the experimental approach is sound, and the results support a useful methodological framework for the in vitro culture of Schistosoma worms and the attainment of sexual maturity, particularly for adult male worms.

We thank the reviewer for highlighting the manuscript’s strengths.

**Reviewer #2 (Public review):**

Summary:

The authors perform confirmation studies of Paul Basch's seminal schistosome work from 1981, demonstrating the development of transformed schistosomules into sexually dimorphic adult parasites, albeit without successful egg production. In addition to the findings from Basch's earlier work, the authors add some new molecular data in the form of an analysis of proliferative cells in in-vitro-derived animals.

Strengths:

The authors successfully confirm experimental results from earlier schistosome researchers, providing a potential new tool for studying schistosome biology without the need for vertebrate hosts.

We thank the reviewer for highlighting the manuscript’s strengths.

Weaknesses:

The display of data from the authors is sometimes difficult to follow/understand where it comes from. For example:

(1) Line 136: The authors claim that parasites in HS and FBS conditions have substantially different mortality rates (11.3 +/- 2.7 vs 5 +/- 2.3) but a quite high p-value (0.8). Analyzing the raw data myself, I obtained a mean of 8.2 +/- 1.7% vs 4.8% +/- 4.3% with a p-value of 0.15. Either the data are not clearly presented, and I did not follow them, or the data presented in the text do not match the raw data in the supplemental files.

We thank the reviewer for pointing this out; we have now edited Supplementary Tables S1 and S6 by turning them into a long format for the sake of clarity. Accordingly, Results, Methods sections, and indicated supplementary tables were edited as follows:

Results, lines 142 ff.:

“No morphological differences were observed between parasites cultured either in FBS or HS within the first week in culture; in both conditions most parasites were classified as early schistosomula [category 1: 76% ± 30 (average ± SD) in FBS and 73% ± 29 (average ± SD) in HS] with few lung (category 2) and early liver schistosomula (category 3) (Figure 1B, week 1; Supplementary Figure S1). The mean mortality (category 0) at week 1 was slightly higher, but not statistically significant (P = 0.42), in worms cultured in HS [9.75% ± 2.76 (average ± SD)] compared to the mortality registered in FBS-cultured parasites [5.52% ± 5.18 (average ± SD), Supplementary Table S6], consistent with previous findings [39].”

Methods, lines 463-465:

“To evaluate differences in mortality between HS- and FBS-cultured parasites, data from 5 experiments were combined and analysed using a Shapiro-Wilk normality test to test normality of the data and a non-parametric Wilcoxon rank sum exact test (Supplementary Tables S1 and S6).”

Supplementary Tables:

Supplementary Table S1. “Raw counts of parasites within each developmental stage category. Each row corresponds to a picture of parasites in culture medium containing FBS or HS. Each column corresponds to the raw parasite counts at indicated stage development (categories 0 to 5), time in culture (Time in days - D), and experimental condition.”

Supplementary Table S6. “Summary of all statistical tests employed in this study. 1. Statistical tests of parasite mortality and the raw data table used for this test. 2. Statistical tests for worm size comparisons (correspond to Figure 2). 3. Statistical tests for worm black gut comparisons (correspond to Figure 3). BG: Black gut. 4. Statistical tests for EdU positive cells comparisons (correspond to Figure 4). Replicate code: E, M and L correspond to day 2, 8 and 15 respectively; R and W correspond to the presence (R) or absence (W) of RBCs added 13 days after transformation.”

For clarity, below we provide the R script used to perform the statistical tests on the data shown in Supplementary Table S6 (column ‘Raw count of parasite developmental category per image and experiment’)

**Author response image 1.**

(2) Line 187/Figure 4: Though it is not clearly stated, it appears that the authors treat their EdU counts as an ordinal data set of 61 steps (from 0 to >60) rather than a continuous measure of EdU+ cells per animal. In this author's opinion, the graph strongly suggests a continuous data set, and the fact that this reviewer had to dig through poorly-labeled raw data to discover the nature of the data is problematic. The authors should either switch to a continuous data set or make it explicit that the data shown are ordinal. If counting EdU+ cells is too arduous, the authors could consider comparing the amount of EdU+ area to the amount of DAPI+ area in maximum intensity projections of their confocal images, as this would roughly approximate the amount of proliferative cells in the animals.

As the reviewer correctly pointed out, the data were treated as ordinal because counting worms with more than 60 Edu+ cells became extremely difficult and highly inaccurate. Therefore, we decided to group in a single category, “60 EdU+ cells”, all worms showing more than 60 EdU+ cells. We have now updated Figure 4 where medians are shown instead of media values, Supplementary Table S5 to provide more comprehensive access to the raw counts, and Supplementary Table S6 to indicate the data for EdU+ cells per worm were considered ordinal. Accordingly, we have revised the corresponding sections as follows:

Results, lines 211 ff:

“HS-cultured schistosomula showed higher numbers of proliferating stem cells, with a median of >48 and >60 EdU+ cells per worm at days 8 and 15, respectively (Figure 4). On the other hand, most FBS-cultured parasites displayed no more than an average of 20 EdU+ cells per worm (Figure 4).”

Methods, lines 520 ff:

“EdU+ cells per parasite were counted for an average of 100 parasites across three independent experiments (Supplementary Table S5). Worms were grouped based on the number of cells per individual, but all those showing ⪰ 60 EdU+ cells were counted in the same group named ‘60 EdU+ cells'. Therefore, the data were considered ordinal data. Statistical analysis was performed by Kruskal-Wallis test with Dunn multiple comparison post-hoc test, with P≤0.05 considered significant (Supplementary Table S6).”

Figure 4 legend, lines 830 ff:

“A. Violin plots showing the number of Edu+ cells per worm at indicated time points (2, 8, and 15 days post cercarial transformation) in parasites cultured either in Foetal Bovine Serum (FBS, blue) or Human Serum (HS, light brown). Human Red Blood Cells (hRBCs) were added in the culture at day 13 post cercarial transformation. The small black dots indicate individual worms, and the big black point indicates the median of EdU+ cells per worm. All worms showing ⪰ 60 EdU+ cells were counted and clustered together in the group named ‘60 EdU+ cells’. Hence, the data were treated as ordinal and statistical analysis performed by Kruskal-Wallis test with Dunn multiple comparison post-hoc test, with P≤0.05 (*) considered significant (Supplementary Tables S5 and S6).”

We thank the reviewer for the very interesting suggestion to quantify cell proliferation by calculating the ratio between EdU+ area to DAPI+ area in maximum intensity projections images. Measuring the fluorescence area for each worm in maximum projection is an excellent idea; however, due to the number of EdU+ cells present in some samples, we think this technique would not provide additional information or produce more detailed data compared with our analysis when the number of Edu+ cells exceeds 60 per worm. We will certainly consider this approximation for future studies.

There are some minor issues as well:

(1) Line 122: It is perhaps incorrect to refer to humans as "the" definitive host of schistosomes, as S. japonicum is primarily considered a zoonotic infection with water buffalo/cows being the primary definitive host.

We thank the reviewer for pointing this out; we have now replaced ‘schistosomes’ with ‘*Schistosoma mansoni*’ (current line 131)

(2) Line 185/298: The authors refer to EdU pulse-chase experiments, but the experiments described here are EdU pulse experiments.

This is a very good point, we thank the reviewer for bringing this up and have accordingly edited by replacing ‘EdU pulse-chase’ with ‘EdU pulse’ experiments in lines 37, 204, and 321.

**Reviewer #3 (Public review):**

Summary:

This study is significant as it established a protocol for the long-term culture of Schistosoma mansoni newly transformed cercariae, which developed in vitro into sexually dimorphic forms. The impact of two different sera, Fetal Bovine Serum (FBS) and Human Serum (HS), added to the culture medium supplemented with human red blood cells was evaluated. The authors demonstrated that HS-cultured parasites were able to digest red blood cells, a critical step for long-term parasite development. Furthermore, while most FBS-cultured parasites did not progress beyond an early liver stage, sexual dimorphism was clearly evident in the HS-cultured worms, albeit delayed compared to in vivo development.

Strengths:

This study could contribute to further in vitro studies for a better understanding of the unique sexual biology of Schistosoma mansoni and for screening novel schistosomicidal compounds. By increasing parasite development in in vitro studies, this protocol could have a positive impact on the principles of the 3Rs (Replacement, Reduction and Refinement) for animal research.

We thank the reviewer for highlighting the manuscript’s strengths.

Weaknesses:

As the authors mentioned, "pairing between male and female parasites was rare. Pairing was observed in approximately ~7% of the experiments, usually after day ~ 80 in culture. Egg production was also not achieved with this protocol.

Following the reviewer’s point and to clarify a misleading point, we have now decided to remove the value of 7% - which corresponds to the percentage of experiments in which couples were observed. However, this value does not accurately reflect the actual number of observed worm pairs, and it is probably misleading. We have updated the text as follows:

Results, lines 230 ff:

“While the establishment of sexual dimorphism was robust and reproducible across more than 15 independent experiments, pairing between male and female parasites was rare. Pairing was observed only in experiments lasting more than 80 days in which we were only able to observe a few couples. In addition, these pairings were temporary (Figures 6A, B; Supplementary Video S4).”

**Recommendations for the authors:**

**Reviewer #1 (Recommendations for the authors):**

The manuscript is well-written overall. However, there are some minor revisions that would further improve the clarity and presentation of the data.

(1) At the beginning of the manuscript, it would be helpful to clearly state three to four specific aims or objectives. This would help readers better understand the expected outcomes and the broader methodological contribution of the study.

We agree with the reviewer and accordingly have stated the overall goals of the study, as follows:

Introduction, lines 106 ff:

“We aimed at optimising a platform to study intra-mammalian schistosomes that supports in vitro sexual dimorphism establishment, consequently leading to an overall positive impact in the 3Rs (Reduction, Replacement, Refinement) for animal research (https://nc3rs.org.uk/) [42]”.

(2) In the abstract, you highlighted the relevance of the work according to the 3R principles of reduction in animal experimentation. However, this point is not clearly introduced in the Introduction section. Including a short discussion of this aspect would improve continuity and context.

Following this and previous item raised by the reviewer, we have now clarified the potential impact in the 3Rs by our research outcomes and included that link to the NC3Rs website and a representative reference [Louis-Maerten E, Rodriguez Perez C, Cajiga RM, Persson K and Elger BS (2024). Conceptual foundations for a clarified meaning of the 3Rs principles in animal experimentation. Animal Welfare, 33, e37, 1–11].

(3) In line 43, please italicize Schistosoma spp.

Edited accordingly.

(4) When discussing the importance of "interfering with sexual development," in line 52, please specify the life cycle stages being referred to.

Revised accordingly as follows:

Introduction, lines 54-56:

“This suggests that interfering with the sexual development of schistosome intra-mammalian stages could potentially restrict human pathology.”

(5) Between lines 56-58, please rephrase this sentence for clarity.

We thank the reviewer for this editorial suggestion. The text has been revised as follows:

Introduction, lines 58 ff :

“Therefore, novel control strategies are urgently needed, and new targets for drug/ vaccine development became a priority. A better understanding of the mechanisms underlying schistosome development, including sexual dimorphism establishment, will pave the wave to achieve this goal.”

(6) In lines 66-68 & line 88, please clarify whether the transcriptomic studies cited were performed in vivo, in vitro, or ex vivo, and indicate the developmental stages analyzed.

We have now included the information suggested by the reviewer as follows:

Introduction, lines 69-70:

“Transcriptomic studies, at both bulk [7-11] and single cell [12-1]4 levels for intra mammalian stages in vivo and ex vivo,...”

(7) Please indicate, in line 110, the day of culture for reference. Without this information, the conversion rates per life cycle stage are difficult to interpret and reproduce. Overall, please try to give an overview in the text of these rates of conversion for context, wherever possible.

Following the reviewer’s question, we have clearly indicated the in vitro and in vivo timings for ‘conversion’ (understood as sexual dimorphism establishment.) We have written:

Introduction, lines 117-120:

“Finally, while most of the FBS-cultured parasites did not progress beyond lung and early liver stage, HS-cultured parasites reached sexually dimorphic stages by week 6, albeit at a slightly delayed rate compared to in vivo development. In the mouse model, parasites become dimorphic by day 21 post-infection (~3 weeks) [12].”

(8) The section beginning with "Furthermore, phenotypic...cell proliferation" (line 110) may be easier to follow if moved earlier in the Introduction.

Following the reviewer’s suggestion, we have moved and slightly rewritten the sentence to current line 112, as follows: “First, phenotypic differences between FBS- and HS- cultured parasites became evident as early as 48 hours in culture, with HS-cultured parasites exhibiting higher rates of cell proliferation resulting in larger worms in the HS condition.”

(9) In line 126, please remove the DOI and add the citation.

Edited accordingly.

(10) When referring to 10-week-old parasites, in line 130, please indicate the developmental stage at which they stalled and relate this to the phenotypic scoring shown in Figure 1.

Based on this suggestion, we have now revised the third paragraph of Results section (‘Sexually dimorphic schistosomes developed entirely in vitro from cercariae’), as follows:

Results, lines 137 ff.:

“The development of schistosomula derived from mechanically transformed cercariae was assessed in at least 15 independent experiments, five of which were maintained over a period of at least 10 weeks to assess parasite survival and ability to mate and produce fertile eggs (Figure 1A; Supplementary Table S1).”

Lines 151 ff.:

“Differences in parasite development between the two conditions became apparent by week 2 (Figure 1B). At this time point, 14.8% ± 24.9 (average ± SD, excluding dead worms) or 36% ± 33.6 (average ± SD, excluding dead worms) of the parasites cultured in FBS or HS, respectively, have reached category 3, i.e., early liver schistosomulum. Parasites in FBS rarely progressed beyond this stage during the 10-week experiment, with very few parasites (<0.1% ± 0.2, average ± SD) reaching category 4, i.e., late liver schistosomulum. In contrast, worms cultured in HS developed over time across all categories, achieving marked sexual dimorphism by week 6 (13.4% ± 18.6, average ± SD) (Figure 1B; Supplementary Figure S3A), as confirmed by PCR (Supplementary Figure S3B; Supplementary Table S2). No differences in the timing for sexual dimorphism establishment were observed between male and female parasites. The mortality rate of FBS-cultured parasites reached an average of 76.24% ± 23.46 (average ± SD) by week 10, after which the experiments under this condition were stopped as most parasites were dead (Supplementary Figure S2). From that time point onwards only parasites in HS were kept in culture. As previously described for the in vivo development of schistosomes [12], in vitro cultured parasites showed developmental asynchrony in agreement with Basch’s observations [33]; however, by week 10 most of the worms in HS (73.7% ± 25.4, average ± SD) acquired an evident sexual dimorphism (Figure 1B).”

(11) In line 142, please provide a standard deviation value for the reported average of 14.8%, if available. As well as the absolute numbers of these parasites or indicate them in the supplementary. Otherwise, it is difficult to understand the true conversion rate.

We followed the reviewer’s suggestions and have now rewritten the text (see above, item 10). In addition, Supplementary Table S1 was edited in long format (see answer for item 1, reviewer #2)

(12) Please explain, IN line 144, why all cultures were maintained for 10 weeks and provide the rationale for this experimental design.

We thank the reviewer for this opportunity to clarify this point and hence improve the manuscript. The experimental condition stopped at week 10 included only FBS-cultured worms, not HS-cultured parasites. This is relevant as most of the parasites in FBS were dead by this time, unlike the HS-developed schistosomes. Indeed, some experimental groups consisting of parasites cultured in HS were maintained for up to 22 weeks. We have now updated the text to clarify this point, as follows:

Results, lines 160 ff.:

“The mortality rate of FBS-cultured parasites reached an average of 76.24% ± 23.46 (average ± SD) by week 10, after which the experiments under this condition were stopped as most parasites were dead (Supplementary Figure S2). From that time point onwards only parasites in HS were kept in culture.”

(13) In lines 146-151, please streamline the timelines of culture conditions and observed outcomes in FBS versus HS media. As the current wording makes interpretation difficult.

Following the reviewer’s suggestion we have streamlined the culture timelines and observed outcomes, as follows:

Results, lines 137 ff.:

“The development of schistosomula derived from mechanically transformed cercariae was assessed in at least 15 independent experiments, five of which were maintained over a period of at least 10 weeks to assess parasite survival and ability to mate and produce fertile eggs (Figure 1A; Supplementary Table S1).”

Results, lines 151 ff.:

“Differences in parasite development between the two conditions became apparent by week 2 (Figure 1B). At this time point, 14.8% ± 24.9 (average ± SD, excluding dead worms) or 36% ± 33.6 (average ± SD, excluding dead worms) of the parasites cultured in FBS or HS, respectively, have reached category 3, i.e., early liver schistosomulum. Parasites in FBS rarely progressed beyond this stage during the 10-week experiment, with very few parasites (<0.1% ± 0.2, average ± SD) reaching category 4, i.e., late liver schistosomulum. In contrast, worms cultured in HS developed over time across all categories, achieving marked sexual dimorphism by week 6 (13.4% ± 18.6, average ± SD) (Figure 1B; Supplementary Figure S3A), as confirmed by PCR (Supplementary Figure S3B; Supplementary Table S2). No differences in the timing for sexual dimorphism establishment were observed between male and female parasites. The mortality rate of FBS-cultured parasites reached an average of 76.24% ± 23.46 (average ± SD) by week 10, after which the experiments under this condition were stopped as most parasites were dead (Supplementary Figure S2). From that time point onwards only parasites in HS were kept in culture. As previously described for the in vivo development of schistosomes [12], in vitro cultured parasites showed developmental asynchrony in agreement with Basch’s observations [33]; however, by week 10 most of the worms in HS (73.7% ± 25.4, average ± SD) acquired an evident sexual dimorphism (Figure 1B).”

(14) In lines 153-159, please clarify comparisons between worms cultured in FBS and HS at equivalent time points (e.g., 2 weeks FBS vs 2 weeks HS), rather than comparing only 10 week cultures.

Following the reviewer’s comment, we have now rewritten the whole third paragraph in Results, under the heading “Sexually dimorphic schistosomes developed entirely in vitro from cercariae” - changes detailed in answers to items 10 and 13 (above).

(15) It would also be helpful to include information on male versus female development in the context of sexual dimorphism.

This is a relevant point that we have not clarified in the original submission - we have now indicated in the text that no differences were detected in the timing for male and female dimorphism establishment. New text included as follows:

Results, lines 159-160:

“No differences in the timing for sexual dimorphism establishment were observed between male and female parasites.”

(16) In line 163, please resolve the editing marks and punctuation.

Resolved accordingly.

(17) In lines 169 and 172, when referring to stages such as "early liver stage," please indicate the corresponding time in culture (e.g., 3 weeks, 7 weeks + 3 days), or define these stage classifications earlier in the manuscript.

Following the reviewer’s suggestion we have now included the developmental category after stating ‘early liver stage’, as follows:

Results, line 187:

“Even though few parasites in FBS reached the early liver stage (category 3)…”

(18) Please indicate, in line 173, the developmental stage of worms used when assessing hRBC digestion in HS and FBS cultures. Additionally, here, it would be useful to discuss how hRBC supplementation may influence worm development beyond culture conditions, including possible molecular mechanisms. As a revision, that way maybe you can include data, if already performed or conduct it, to show the effect of adding or not adding hRBC even in HS cultured worms.

We thank the reviewer for highlighting this important item that warrants further clarification. As stated in Results washed human red blood cells (hRBCs) were added to the culture at day 13. Pilot experiments in which hRBCs were added at different time points had been previously performed; no hemoglobin digestion was apparent when hRBCs were added at days 4, 5 and 6 consistent with previous findings (Correnti JM, Jung E, Freitas TC, Pearce EJ. Transfection of Schistosoma mansoni by electroporation and the description of a new promoter sequence for transgene expression. Int J Parasitol. 2007 Aug;37(10):1107-15. doi: 10.1016/j.ijpara.2007.02.011. Epub 2007 Mar 18. PMID: 17482194.).

Following this observation, we have added a line to clarify this point, as follows (lines 181187): “Based on both previous reports [45], and pilot experiments in which adding human Red Blood Cells (hRBCs) to the culture before day ~10 did not show obvious haemoglobin digestion, we decided to supplement the culture media with hRBCs at day 13. The addition of hRBCs allowed the parasites to feed and thus continue their development [19]. At this point, they began to swallow and degrade erythrocytes, producing hemozoin, a black pigment derived from host haemoglobin degradation and visible in the worms' intestines.”

Regarding the specific effect of adding hRBCs in the culture, this is a very good point. First, it has been well established for more than four decades that schistosomes need red blood cells in culture to grow, as example see (Basch, P. F. Cultivation of *Schistosoma mansoni* in vitro. II. production of infertile eggs by worm pairs cultured from cercariae. J Parasitol 67, 186-190 (1981); Basch, P. F. Cultivation of *Schistosoma mansoni* in vitro. I. Establishment of cultures from cercariae and development until pairing. J. Parasitol. 67, 179-185 (1981)). Second, we are currently analysing transcriptomic data from parasites cultured in different conditions, including in the presence or absence of hRBCs. We decided not to include these data and analyses in the current manuscript, as they fall outside its scope.

(19) In line 183, please clarify whether the referenced single-cell transcriptomic data were obtained from adult worms.

We have now clarified this point in the manuscript as follows:

Results, lines 199 ff:

“In schistosomes, a complex stem cell system consisting of both somatic and germline stem cells has been described by leveraging recent single cell transcriptomic data across different developmental stages, including schistosomula and adult worms [47].”

(20) In lines 210 and 213, please indicate the absolute number of worms used for these observations, rather than only percentages. If possible, also report any sex bias in pairing.

Following this and a similar item raised by reviewer #3 (public review), we decided to remove the mention of 7% given it is misleading. This percentage corresponds to the percentage of experiments in which couples were observed. However, this value does not accurately reflect the actual number of observed worm pairs, and it is probably misleading. We have updated the text as follows:

Results, lines 230 ff.:

“While the establishment of sexual dimorphism was robust and reproducible across more than 15 independent experiments, pairing between male and female parasites was rare. Pairing was observed only in experiments lasting more than 80 days in which we were only able to observe a few couples. In addition, these pairings were temporary (Figures 6A, B; Supplementary Video S4).”

(21) In the final results section, please clarify whether pairing enhances sexual maturation of already mature worms or whether maturation occurs primarily after pairing.

This is a very relevant point, and we thank the reviewer for giving us the opportunity to clarify it in the manuscript. As described in the manuscript the parasite sexual dimorphism was established in vitro and developed male and female parasites were capable of pairing. Moreover, enlarged oocytes in the ovary’s posterior section of in vitro developed female parasites became apparent after pairing. This observation (Figure 6E, F and Supplementary Video S6) suggests that these female parasites, fully developed in HS-supplemented culture media, were not only capable of pairing, but of starting to fully maturate. We have clarified this aspect in the manuscript as follows:

Results, lines 243 ff.:

“Moreover, in vitro developed females coupled with ex vivo collected mature males displayed signs of primordial ovary maturation with larger oocytes towards the posterior region of the ovary (Figure 6E, F; Supplementary Video S6). On the other hand, females developed in vitro but not paired with ex vivo collected males remained immature.”

(22) Further in the Materials and methods sections, please clarify, isn't 8000 schistosomula/well of a 6-well plate really a confluent culture condition, and does it contribute to NTS mortality in that way, as shown in previous in vitro transformation publications? Please clarify, at least with relative values, percentages of parasite transformation in such a concentrated system.

No formal titration experiments were carried out but based on empirical observations during pilot experiments we decided to add no more than 8,000 schistosomula per well. This is something to further investigate in the future. We have now added the following sentence in Methods:

Methods, lines 423-426:

“The number of parasites cultured per well (~8,000 schistosomula) was determined empirically, as no formal titration experiments were performed. At higher densities (>10,000 per well), more frequent media changes were required, and parasite development appeared to be impaired.”

(23) Also, what was the rationale of adding hRBCs as early as 13 days post-transformation, when the parasites are in the lung and early liver stage, just forming the guts? Therefore, is it possible that this would have contributed to the observation of lesser parasites disgesting hRBCs? Also, were the hRBC supplemented each time with the media change? This was not clear.

We thank the reviewer for these questions. The rationale of adding hRBCs at day 13 has been elaborated above (question 18). In addition, in the mouse model, parasites have already migrated through and left the lungs by day 13 post-infection, as described by Nation et al [Nation CS, Da’dara AA, Marchant JK, Skelly PJ (2020) Schistosome migration in the definitive host. PLoS Negl Trop Dis 14(4): e0007951] as follows: “In the mouse, *S. mansoni* schistosomula begin to arrive in the lungs between 2 and 3 days post-infection, peaking at around day 7 and lasting until around day 11”. Hence, we do not think that adding hRBCs at day 13 contributed to the observation of fewer parasites digesting hemoglobin, because this was only seen in parasites cultured in FBS, not in HS.

The hRBCs were replaced every two weeks, or sooner if their numbers decreased due to consumption. We have now clarified this point in Methods as follows (lines 427-430): “LTC medium was replaced twice a week and washed human red blood cells (hRBCs) added to a final concentration of 0.02% v/v at 13 days after transformation. Washed hRBCs were replaced every two weeks, or sooner if their numbers decreased due to consumption.”

(24) In the Discussion, please address the limitations related to the relatively late onset and low frequency of pairing in vitro.

Following the reviewer’s suggestion and comments from reviewer #1, we have now included a section in Discussion highlighting the limitations of the study and avenues to overcome these in the future.

Discussion, line 360 ff.:

“Considering these elements in future experiments will help overcome the limitations encountered in this study, including the low rate of spontaneous pairing between in vitro– developed male and female worms and the requirement for extended culture periods (>70 days). In addition, further research is needed to assess the role of host- and parasite-derived cues in schistosome development.”

(25) Figure 1: Please consider adding arrows or markers indicating which parasites correspond to the representative developmental stages used for classification.

We acknowledge the reviewer for the suggestion; however, we respectfully consider this may not be necessary as (1) the images shown in Figure are representative pictures of each time point included for illustrative purposes; (2) Supplementary Figure S1 clearly depicts representative images of worms in each developmental category associated with specific morphological descriptions. For greater clarity we have now added the following text at the end of Figure 1 legend:

Figure 1 legend, line 810-811:

“A detailed description of the developmental categories and representative images are provided in Supplementary Figure S1.”

(26) Figure 2: This plot is somewhat misleading in showing that the HS cultured worms grew significantly more than the FBS worms, where the latter did not grow at all, as also shown by the blue bars all over the plot.

We appreciate the reviewer’s observation; critically, the data shown in Figure 2 represent measurements of the worm's area, which means that some worms may have become longer but thinner maintaining the same area. Most of the FBS-cultured worms did not develop beyond lung or early liver stages, in which the parasites were long/ thin or shorter/wide, respectively. Therefore, the overall area of these FBS-cultured worms almost did not change (please see the raw data and statistical analyses in Supplementary Tables S3 and S6). We believe that, as presented, Figure 2 is sufficiently clear and self-explanatory. However, we would be happy to consider any suggestions to further clarify this point in the manuscript.

(27) Figure 3: For panel A, what is the worm percentage corresponding to? The context is missing. Please clarify in the text.

Following the reviewer’s question and for clarity, we have now (1) modified the axis-legend in Figure 3 as “Percentage of worms displaying or not Black Guts - BG (%)”, and (2) slightly edited the legend as follows:

Figure 3 legend, lines 820-823:

“Bar Plot representing the percentage of Human Serum (HS)- or Foetal Bovine Serum (FBS)-cultured schistosomula with (blue bar) or without (light brown bar) black guts (BG) due to the presence of intestinal hemozoin.”

**Reviewer #2 (Recommendations for the authors):**

The authors need to clarify their presentation of data. The raw data needs to be more clearly labeled/explained, and the representation of the data in Figure 4A needs to be explicitly described or changed.

We acknowledge the reviewer for highlighting this issue related with the data presentation and have decided to follow their advice by editing Figures 3 and 4, and improving the data presentation in Supplementary Tables S1, and S4-S6. In particular:

Figure 3. We have now modified the axis-legend as “Percentage of worms displaying or not Black Gut - BG (%)”, and slightly edited the legend as follows:

Figure 3 legend, lines 820-823:

“Bar Plot representing the percentage of Human Serum (HS)- or Foetal Bovine Serum (FBS)-cultured schistosomula with (blue bar) or without (light brown bar) black guts (BG) due to the presence of intestinal hemozoin.”

Figure 4. We have edited this figure to show medians instead of media values, and updated the legend as follows: lines 830 ff.:

“A. Violin plots showing the number of Edu+ cells per worm at indicated time points (2, 8, and 15 days post cercarial transformation) in parasites cultured either in Foetal Bovine Serum (FBS, blue) or Human Serum (HS, light brown). Human Red Blood Cells (hRBCs) were added in the culture at day 13 post cercarial transformation. The small black dots indicate individual worms, and the big black point indicates the median of EdU+ cells per worm. All worms showing ⪰ 60 EdU+ cells were counted and clustered together in the group named ‘60 EdU+ cells’. Hence, the data were treated as ordinal and statistical analysis performed by Kruskal-Wallis test with Dunn multiple comparison post-hoc test, with P≤0.05 (*) considered significant (Supplementary Tables S5 and S6).”

Supplementary Table S1. We have clarified the data presentation by turning it into a long format and updated the legend accordingly as follows (lines 864-867): “Raw counts of parasites within each developmental stage category. Each row corresponds to a picture of parasites in culture medium containing FBS or HS. Each column corresponds to the raw parasite counts at indicated stage development (categories 0 to 5), time in culture (Time in days - D), and experimental condition.”

Supplementary Table S4. We have clarified the table by turning it into a long format, simplified the data presentation, and updated the legend accordingly as follows (lines 873874): “Percentage of parasites displaying either black positive (hemozoin) or black negative (no hemozoin) intestine.”

Supplementary Table S5. We have simplified the table by turning it into a long format, and explained the naming for elements in columns C (‘Group’) and D (‘Replicate’). We have updated the legend accordingly as follows (line 876 ff.): “Raw counting of EdU positive cells per parasite for indicated experimental group, replicate and experiment in long format. The worms were classified by group (column C) and replicate (column D), using the following code: E (‘early’), M (‘medium’) and L (‘late’), corresponding to days 2, 8 and 15, respectively. R and W correspond to conditions with (R) or without (W) human red blood cells, and HS and FBS to culture medium employed.”

Supplementary Table S6. We have incorporated a new section with the statistical analyses for parasite mortality estimation and updated the legend accordingly as follows (lines 882887): “Summary of all statistical tests employed in this study. 1. Statistical tests of parasite mortality and the raw data table used for this test. 2. Statistical tests for worm size comparisons (correspond to Figure 2). 3. Statistical tests for worm black gut comparisons (correspond to Figure 3). BG: Black gut. 4. Statistical tests for EdU positive cells comparisons (correspond to Figure 4). Replicate code: E, M and L correspond to day 2, 8 and 15 respectively; R and W correspond to the presence (R) or absence (W) of RBCs added 13 days after transformation.”

**Reviewer #3 (Recommendations for the authors):**

The study was well conducted, and the data presented clearly support the conclusions. The protocol is well described, making it reproducible. The pairing experiments could be improved.

Specific Questions.

(1) "Male and female adult worms that developed in vivo and recovered from mice by portal perfusion on day 42 post-infection were sorted by sex and placed in culture with worms of the opposite sex developed in vitro (>70 days). Within 24 hours of initiating the co-culturing of in vitro developed worms with ex vivo collected worms, couples were observed".

In the interest of clarity, and considering that stating ‘worms developed in vivo were collected from infected mice’ is redundant, we have now shortened and edited these lines as follows (lines 238- 242): “Male and female adult worms were recovered from mice by portal perfusion on day 42 post-infection, sorted by sex and placed in culture with worms of the opposite sex developed in vitro. Within 24 hours of initiating the co-culturing of in vitro developed worms with ex vivo collected worms, couples were observed (Figures 6C, D; Supplementary Video S5).”

(2) Have the authors conducted experiments with in vitro female and male parasites under the same experimental conditions as the in vitro/ex vivo pairing experiments? Is it possible that the tissue culture medium used for the development of sexually dimorphic forms is inhibiting pairing?

The reviewer raises an interesting point that warrants clarification. First, the experimental conditions tested for in vitro developed parasites were the same as for the pairing experiments, as the ex vivo collected worms were washed and placed in HS-supplemented media. Second, as the culture conditions were the same (same culture protocol and medium) between in vitro pairing and in vitro / ex vivo pairing experiments, we do not think that the tissue culture medium used for developing sexually dimorphic parasites inhibited the pairing. As elaborated in Discussion (see below), key factors, probably derived from the host, are missing in the in vitro system explaining the low rate of spontaneous pairing between in vitro developed, sexually dimorphic male and female worms. This was discussed as follows (lines 340-343): “That said, while our system was highly efficient in producing sexually dimorphic worms, spontaneous pairing between male and female parasites was extremely rare, mainly in aged in vitro cultures (from 80 to 100 days in culture) indicating that other factors, e.g., cholesterol, may be missing [35].”
